# Intra-colony venom diversity contributes to maintaining eusociality in a cooperatively breeding ant

**DOI:** 10.1186/s12915-022-01507-9

**Published:** 2023-01-08

**Authors:** Samuel D. Robinson, Vanessa Schendel, Christina I. Schroeder, Sarah Moen, Alexander Mueller, Andrew A. Walker, Naomi McKinnon, G. Gregory Neely, Irina Vetter, Glenn F. King, Eivind A. B. Undheim

**Affiliations:** 1 Centre for Advanced Imaging, The University of Queensland, St Lucia, QLD 4072 Australia; 2 Institute for Molecular Bioscience, The University of Queensland, St Lucia, QLD 4072 Australia; 3 Present Address: Genentech, 1 DNA Way, South San Francisco, CA 94080 USA; 4 Dr. John and Anne Chong Lab for Functional Genomics, Charles Perkins Centre, Centenary Institute, and School of Life and Environmental Sciences, University of Sydney, Sydney, NSW Australia; 5 School of Pharmacy, The University of Queensland, Woolloongabba, QLD 4102 Australia; 6 Centre for Ecological and Evolutionary Synthesis, Department of Biosciences, University of Oslo, 0316 Oslo, Norway

**Keywords:** Formicidae, Kin selection, Group selection, Eusocial, Peptide, Toxin

## Abstract

**Background:**

Eusociality is widely considered to evolve through kin selection, where the reproductive success of an individual’s close relative is favored at the expense of its own. High genetic relatedness is thus considered a prerequisite for eusociality. While ants are textbook examples of eusocial animals, not all ants form colonies of closely related individuals. One such example is the ectatommine ant *Rhytidoponera metallica*, which predominantly forms queen-less colonies that have such a low intra-colony relatedness that they have been proposed to represent a transient, unstable form of eusociality. However, *R*. *metallica* is among the most abundant and widespread ants on the Australian continent. This apparent contradiction provides an example of how inclusive fitness may not by itself explain the maintenance of eusociality and raises the question of what other selective advantages maintain the eusocial lifestyle of this species.

**Results:**

We provide a comprehensive portrait of the venom of *R. metallica* and show that the colony-wide venom consists of an exceptionally high diversity of functionally distinct toxins for an ant. These toxins have evolved under strong positive selection, which is normally expected to reduce genetic variance. Yet, *R. metallica* exhibits remarkable intra-colony variation, with workers sharing only a relatively small proportion of toxins in their venoms. This variation is not due to the presence of chemical castes, but has a genetic foundation that is at least in part explained by toxin allelic diversity.

**Conclusions:**

Taken together, our results suggest that the toxin diversity contained in *R. metallica* colonies may be maintained by a form of group selection that selects for colonies that can exploit more resources and defend against a wider range of predators. We propose that increased intra-colony genetic variance resulting from low kinship may itself provide a selective advantage in the form of an expanded pharmacological venom repertoire. These findings provide an example of how group selection on adaptive phenotypes may contribute to maintaining eusociality where a prerequisite for kin selection is diminished.

**Supplementary Information:**

The online version contains supplementary material available at 10.1186/s12915-022-01507-9.

## Background

Eusocial animals form colonies that are organized into reproductive and non-reproductive castes from multiple overlapping generations of individuals that care cooperatively for the young. Ants are among the best-known examples of eusociality, with colonies typically formed by a single reproductive female (“queen”) that is assisted by her numerous non-reproductive daughters (workers, soldiers, etc.). While this may seem like an altruistic act on the part of the non-reproductive workers, the close genetic relationship within the colony means that any success of the colony also benefits the workers’ inclusive fitness, i.e., the sum of direct dispersal of their genes and (indirect) dispersal of their genes through close relatives. This evolutionary strategy, known as kin selection, is considered to be among the primary forces that maintain eusociality [1].

While genetic relationship is the main driver in evolution by kin selection, there are also selection forces that diminish the benefit of close kinship in eusocial colonies. In ants with a monogynous (single queen) colony structure, increased intra-colony genetic diversity is usually achieved by polyandry (mating with multiple males), and the resulting increased genetic diversity has been shown to be beneficial to colony fitness in the harvester ant *Pogonomyrmex occidentalis* [2]. In the leaf-cutting ant *Acromyrmex echinatior*, groups of ants with more genetically diverse patrilines were more resistant to the entomopathogenic fungus *Metarhizium anisopliae* [3]. There are also many species of ants that deviate from the monogynous (single queen) colony structure and form colonies containing multiple queens, or in some cases mated fertile workers called gamergates. In most cases, these all contribute to both the non-reproductive and reproductive castes, suggesting the cost of reduced kinship is outweighed by the benefits of increased genetic diversity. However, there is variable support for how beneficial this increased genetic diversity is, or what these benefits may be [3].

The Australian “green” or “greenhead” ant, *Rhytidoponera metallica*, is an extreme example of an ant that forms colonies that deviate from the single-queen monogynous structure: the vast majority of *R. metallica* colonies lack a queen caste, and instead 5–15% of workers in a colony mate and produce offspring [4]. In addition, most new colonies are formed by “budding” as opposed to by dispersal of winged reproductive females, and hence genetic dispersal is primarily done by winged males. These males generally mate only once within each colony, meaning the offspring of different gamergates are usually from independent patrilines [5]. This reproductive strategy results in some of the lowest known genetic relatedness among nestmates of any eusocial insect [6]—so low that it has been proposed to be an unstable, transient form of eusociality [7]. Nevertheless, *R. metallica* is an extremely successful ant species, measured by its abundance and wide distribution across the Australian subcontinent. This apparent contradiction raises the question of what selective advantages contribute to the maintenance of eusociality in *R. metallica*.

One adaptive trait in which increased diversity could be of benefit to the colony is venom. Venom is among the most frequently evolved adaptations known and has emerged on >100 occasions in the animal kingdom where it facilitates a range of ecological functions, including predation, feeding, defense, and intraspecific competition [8]. Venom is an essential trait for most ant species and plays central roles in both foraging and colony defense. Most ant venoms are composed primarily of peptide toxins that belong to a single gene superfamily called aculeatoxins [9–12]. The aculeatoxins have undergone extensive functional and structural diversification that can be tied to their diverse ecological roles [9], meaning their composition in the venom can be used to infer ecological function.

Here, we provide a comprehensive portrait of the venom of the cooperatively breeding ant *R. metallica.* We show that the colony-wide venom of *R. metallica* is extremely diverse for an ant venom and that this diversity is achieved through the contribution of divergent venoms from workers of the same colony. The peptides that dominate the venom of *R. metallica* have evolved under strong positive selection to achieve multiple distinct functions, which are likely reflected as differences in the functional ecology of different workers. We discuss the mechanisms that may result in this colony-wide venom diversity and propose that phenotypic diversity in adaptive traits could itself explain the maintenance of eusociality in low-kinship colonies by enabling exploitation of wider sets of natural resources and defense against a wider range of predators.

## Results

### The venom of *R. metallica* is rich in peptide toxins

**Fig. 1:**
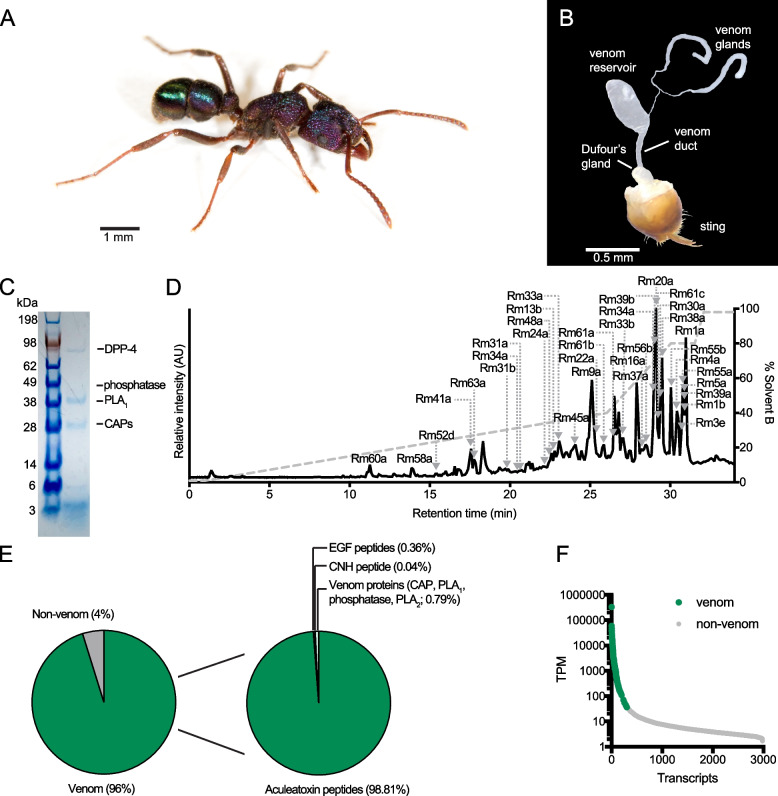
The polypeptidic venom composition of *R. metallica*. **A ***R. metallica* worker. **B** Dissected venom apparatus of *R. metallica* worker. **C** SDS-PAGE gel of *R. metallica* venom (pooled) labeled with major venom proteins identified by LC-MS/MS. **D** Total ion chromatogram from LC-MS/MS analysis of *R. metallica* venom (pooled from ~100 worker ants) with peaks corresponding to identified venom peptides labeled. Additional peptides were identified only in the reduced and alkylated and/or reduced, alkylated and trypsin-digested venom samples. **E** Venom component-encoding transcripts (i.e., those encoding peptides detected in the venom itself) comprised 96% of total transcript expression. Of these, transcripts encoding aculeatoxin peptides, EGF-domain peptides, a CNH-domain peptide, and venom proteins comprised 98.81, 0.36, 0.04, and 0.79%, respectively, of venom component expression. **F** Venom component-encoding transcripts (highlighted in green) constitute almost all the most highly expressed transcripts. TPM, transcripts per million

We used a combined transcriptomic and mass spectrometry (MS)-based proteomic strategy to generate a full portrait of the polypeptidic venom composition of the greenhead ant, *R. metallica* (Fig. 1A). We collected venom from *R. metallica* by inciting ants to sting a thin layer of parafilm (Additional file 1: Video S1). Unless otherwise stated, the venom samples analyzed in subsequent experiments were venom droplets pooled from ~100 individuals and were thus representative of the colony rather than any given individual. We also sequenced the transcriptome of the venom apparatus (venom gland filaments, venom reservoir (including the convoluted gland), and venom duct; Fig. 1B) dissected and pooled from 30 of these ants. 50,801,844 demultiplexed paired-end reads were generated by Illumina NextSeq RNA sequencing, and following adaptor trimming, quality trimming and filtering and error correction were assembled de novo using Trinity [13]. Assembly yielded a total of 41,698 contigs with an E_90_ value (the number of transcripts that account for 90% of the sequenced reads) of 49, which is indicative of a strong bias towards the expression of relatively few transcripts.

An examination of *R. metallica* venom by SDS-PAGE (Fig. 1C) revealed strong staining at masses <6 kDa indicative of a predominantly peptidic venom. To identify the four bands >20 kDa, we excised the bands and analyzed them by liquid chromatography–tandem mass spectrometry (LC-MS/MS). These corresponded to a dipeptidyl peptidase IV (DPP-4), a phosphatase, a phospholipase-A_1_ (PLA_1_), and several CAPs (cysteine-rich secretory protein, insect venom allergen antigen 5, and pathogenesis-related 1 protein)]. Of these, DPP-4, which is also detected at low levels in other ant venoms [9], is probably not a functional venom component but instead is responsible for the maturation of venom peptides [14]. CAPs, phosphatases, and phospholipases have been reported in other hymenopteran venoms. The function of the former two in venom is unclear, while the PLA_1_ presumably catabolizes phospholipids in cell membranes, possibly enhancing the efficacy or spread of other venom components [15].

We used a combination of top-down and bottom-up sequencing to further examine the polypeptide composition of *R. metallica* venom. LC-MS/MS data of three venom samples (native (Fig. 1D); reduced and alkylated; and reduced, alkylated, and trypsin-digested) were searched against a database comprising the translated venom apparatus transcriptome. We confirmed (by top-down sequencing of native venom) the complete mature sequence of 54 peptides and from top-down data obtained partial sequences for 17 additional venom peptides. An additional 34 peptides that were not detected in our proteomic data were included as probable venom components based on high-sequence similarity and similarly high-expression level estimates to identified venom peptides.

In total, we report 123 polypeptide components in the venom of *R. metallica* (Additional file 2: Table S1). Transcripts encoding these venom components corresponded to 95.8% (958,055 TPM) of all venom apparatus-derived reads (Fig. 1E) and were confined to the high-expression portion of the venom apparatus transcriptome, where they constituted almost all the most highly expressed transcripts (Fig. 1F) i.e., 99 of the 100 most highly expressed transcripts encoded venom components. The 123 venom components can be divided into five general toxin types: 100 cationic and hydrophobic peptides derived from the aculeatoxin gene superfamily [9]; five epidermal growth factor (EGF)-like peptides; one crustacean neurohormone (CNH)-like peptide; four enzymes (a DPP-4, a phosphatase, a PLA_1_, and a PLA_2_), each represented by a single isoform; and several CAPs. Of these, transcripts encoding aculeatoxin peptides account for 98.81% of venom component expression, while the EGF-domain peptides, CNH-domain peptide, and venom proteins account for 0.36, 0.04, and 0.79%, respectively. Thus, in terms of both absolute number and expression level, the venom of *R. metallica* is dominated by peptides derived from the aculeatoxin gene superfamily.

**Fig. 2:**
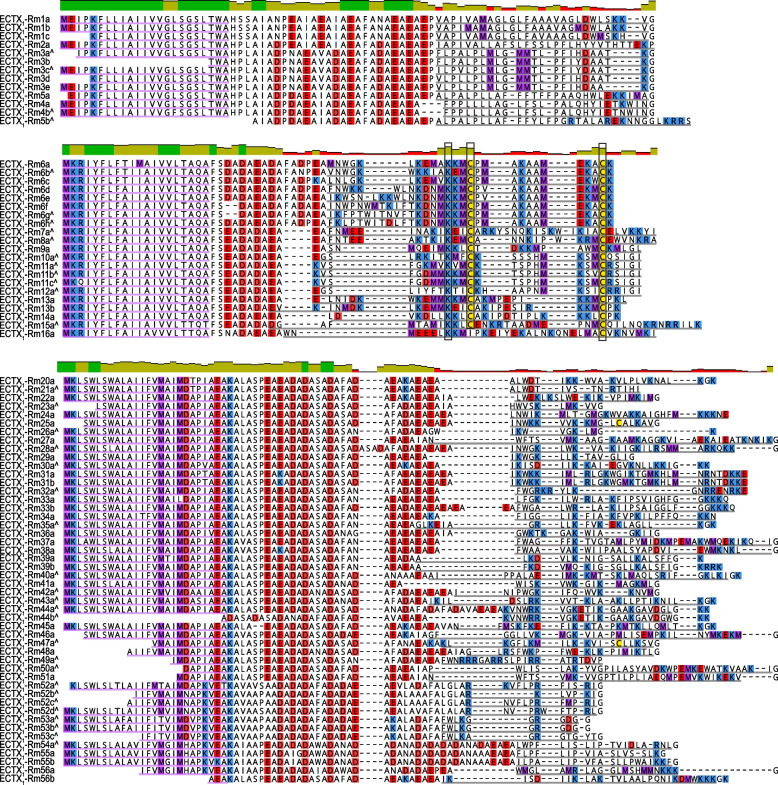
Amino acid sequence alignments of the three major aculeatoxin clades of *R. metallica* venom. Sequence identity plots (sliding window of 2) are shown above each alignment. Methionine, lysine/arginine, aspartate/glutamate, and cysteine residues are highlighted in purple, blue, red, and yellow, respectively. Signal peptides and mature peptides are underlined in purple and gray, respectively. ^ indicates sequences with predicted mature peptide regions.

The mature peptides of *R. metallica* aculeatoxins can be grouped into three “clades” based on general structural properties (Fig. 2). They nevertheless share a set of distinct biophysical features regardless of the presence or absence of cysteine residues: all are rich in lysine and hydrophobic residues, and many have sequence characteristics suggestive of a capacity to form amphipathic helices, such as the interspersal of cationic and hydrophobic residues at frequencies similar to the length of an alpha-helix turn. These are features shared with most aculeatoxin peptides of other aculeate hymenopteran venoms [9]. Three of the *R. metallica* aculeatoxin peptides (ECTX_1_-Rm16a, Rm25a, and Rm47a) each contain a single cysteine residue. Of these, ECTX_1_-Rm16a was confirmed in the native venom as a homodimer. A further 23 (ECTX_1_-Rm6–Rm15 and their isoforms, Rm60a, Rm62a, and Rm63a) contain two cysteine residues. Of these, Rm9a, Rm13b, Rm60a, and Rm63a were detected in the venom as monomers (i.e., the pair of cysteine form an intrachain disulfide bond), with no evidence of dimerization detected.

### Ants from the same colony have distinct venoms

**Fig. 3:**
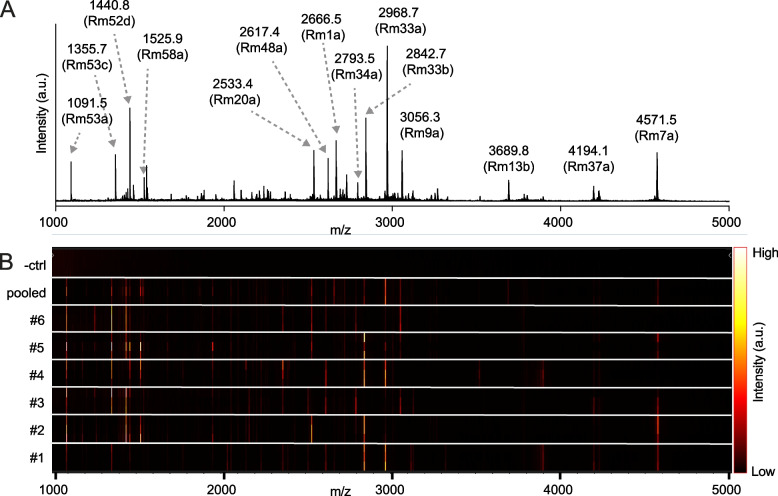
Individual *R. metallica* workers from a single colony have distinct venom compositions. **A** MALDI mass spectrum, generated in reflectron positive mode, of pooled *R. metallica* venom. Selected peaks are labeled with observed monoisotopic MH^+1^. **B** Gel view of MALDI-MS spectra collected in three technical replicates from pooled venom and venoms of six individual workers illustrates intra-colony variation in *R. metallica* venom

The presence of over 100 distinct peptides in the pooled venom of *R. metallica* contrasts with what has been reported of other ant venoms, which are typically less complex [9, 10, 12]. Due to the low relatedness among nestmates in *R. metallica* [6], we next assessed whether or not this genetic heterogeneity was reflected at the level of venom composition and thus contributed to the toxin diversity of our pooled venom. We collected several individual *R. metallica* workers (from the same nest entrance) and, using matrix-assisted laser desorption/ionization (MALDI) MS, compared their venom composition to one another and to the pooled venom (Fig. 3). Analysis of the pooled venom sample by MALDI MS generated a mass profile consistent with the data obtained by ESI MS. 14 major peaks between 1000 and 5000 Da, assigned to identified peptides, are labeled in Fig. 3A. We found that none of the individual worker venoms analyzed were identical, even among the most abundant components, which contrasted the highly similar spectra in our technical replicates (Fig. 3B). Instead, each exhibited a unique subset of the pooled venom components: of the 14 major peaks in the pooled venom, 10, 10, 7, 10, 10, and 8 matching peaks were detected in at least one of the replicate spectra of the venoms from individuals 1, 2, 3, 4, 5, and 6, respectively (Fig. 3B). Each of the six workers produced between 50 and 71% of the most abundant peptides detected in the pooled venom by MALDI-MS. Three peptides (Rm33a, Rm33b, and Rm53a) were detected in all individuals, while two peptides (Rm1a and Rm13b) were detected only in the pooled sample. Thus, the complex venom composition of the *R. metallica* colony is the sum of many simpler but highly variable worker venoms that share an average of just 62% compositional identity.

**Fig. 4:**
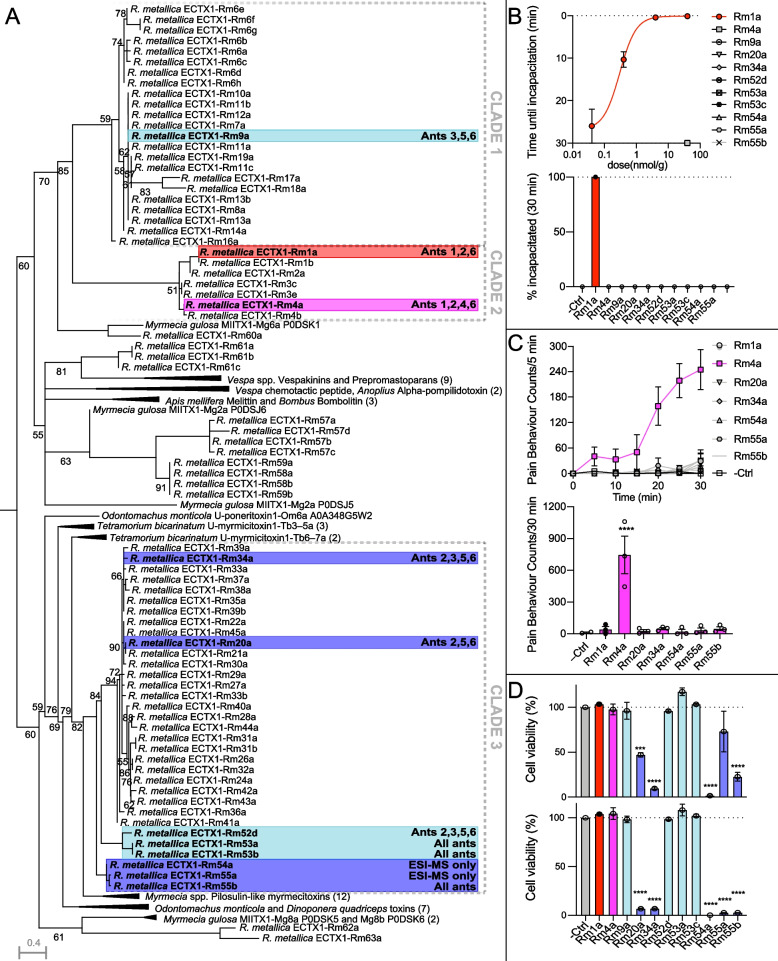
Functional radiation of *R. metallica* aculeatoxins. **A** Phylogenetic analysis showed *R. metallica* aculeatoxins form functionally distinct clades and that individual workers contained representatives from several of these clades. Phylogeny was calculated by maximum likelihood under the JTT+G4 model and is displayed as a midpoint rooted tree. The names of each peptide synthesized and screened for activity are highlighted and shown in bold, with the prevalence of each indicated on the right and the highlight color corresponding to the type of activity detected in b–d; insecticidal (red), defensive (purple), cytolytic (blue), unknown (cyan). **B** Intra-abdominal injection of Rm1a (but not other venom peptides) in crickets (*Acheta domesticus*) caused dose-dependent, irreversible, and lethal incapacitation (*n* = 3 per group). Curves were fitted using a four-parameter Hill equation (variable slope) in Graphpad Prism8 (top). At a dose of 40 nmol/g, only Rm1a resulted in incapacitation of crickets even after 30 min (bottom). **C** Upon intraplantar injection of 200 pmol peptide (*n* = 3 per group), only Rm4a caused spontaneous nocifensive behavior in mice as measured by pain behavior counts in 5-min bins (top) or cumulative counts (bottom) across 30 min. **D** 10 μM Rm20a, Rm34a, Rm54a and Rm55b significantly reduced HAP cell viability; *n* = 3 per group. 100 μM (bottom) Rm55a significantly reduced HAP cell viability to 24.6 ± 8.1% (*n* = 3) and has therefore also been labeled as cytotoxic in panel A. All data are expressed as mean ± SEM. Sequence alignments can be found in Additional file 3, individual datapoints for time courses in B and C are shown in Additional file 4: Fig. S1

To investigate the genetic basis underlying the compositional differences between workers, we reconstructed the evolutionary history of the *R. metallica* aculeatoxins by maximum likelihood (Fig. 4). The resulting tree topology suggests the *R. metallica* aculeatoxins represent six toxin lineages that are taxonomically widespread in Aculeata. Two of these lineages dominate the toxin repertoire of *R. metallica* and form three distinct and functionally diverse clades (clades 1–3; Fig. 4A). Mapping the most abundant toxins onto this toxin gene phylogeny revealed that individual venoms are not biased to certain clades, but instead contain a subset of toxins from each clade. While this pattern could be due to variable expression of closely related paralogues across individuals, it may also be explained by allelic variation: because our transcriptome was sequenced from pooled venom apparatuses from 30 individuals, the apparent gene expansions observed in our phylogenetic tree could represent a mix of paralogues and allelic variants (within-population orthologues).

### Individual venom peptides are tuned for defense or predation

We next examined whether the differences in worker venom composition could translate to differences in venom function. *R. metallica* readily uses its venom defensively, and stings from this species are notoriously painful. Adult workers also use their sting to incapacitate arthropod prey, which they carry back to their colony to feed to the larvae. Because the venom yield from individual workers is insufficient for screening activities that correspond to both of these functions, we therefore selected 11 abundant representative aculeatoxin peptides, which we synthesized by solid-phase peptide synthesis and tested for insecticidal, algogenic, cytotoxic, and antimicrobial activity.

We assessed the insecticidal effects of the synthetic peptides against house crickets (*Acheta domesticus*) and fruit flies (*Drosophila melanogaster*). We found that only one of the eleven peptides tested, Rm1a, was able to incapacitate crickets at a concentration of 40 nmol/g. The dose of Rm1a required to paralyze 50% of crickets 30 min after injection (PD_50_ 30 min) was 0.3 nmol/g, with incapacitation being immediate at 40 and 4 nmol/g and delayed at lower doses (Fig. 4B). No crickets recovered—even the lowest dose tested (40 pmol/g) was ultimately lethal. Similarly, while injection of 5 nmol/g Rm1a caused instant death in 100 ± 0% of flies, no other peptide showed the same effect (mean ± SD; Additional file 4: Fig. S1A). While injection of 5 nmol/g Rm34a, Rm54a, and Rm55b into *D. melanogaster* disrupted motor behavior and caused significant differences compared to control, as little as 50 pmol/g Rm1a paralyzed most flies without killing them (95.8 ± 7.2% paralyzed, 25.0 ± 12.5% dead; Additional file 4: Fig. S1B). Closer testing of the potency of Rm1a revealed that the dose required to kill 50% of flies 5 min after injection (LD_50_ 5 min) was 0.17 nmol/g and the dose required to paralyze 50% of flies within 5 min (PD_50_ 5 min) was 23 pmol/g (Additional file 4: Fig. S1C). Rm1a was abundant in the pooled venom but was only detected at relatively low levels in half of the individual worker venoms examined, indicating it is highly abundant in some worker venoms but absent or only present in very low abundance in others. Although our models for insecticidal activity are not native prey species, the differences observed between the tested toxins, and their variable abundances between individual venoms, suggest that there are likely to be differences in individual worker venom efficacy against different prey taxa.

**Fig. 5:**
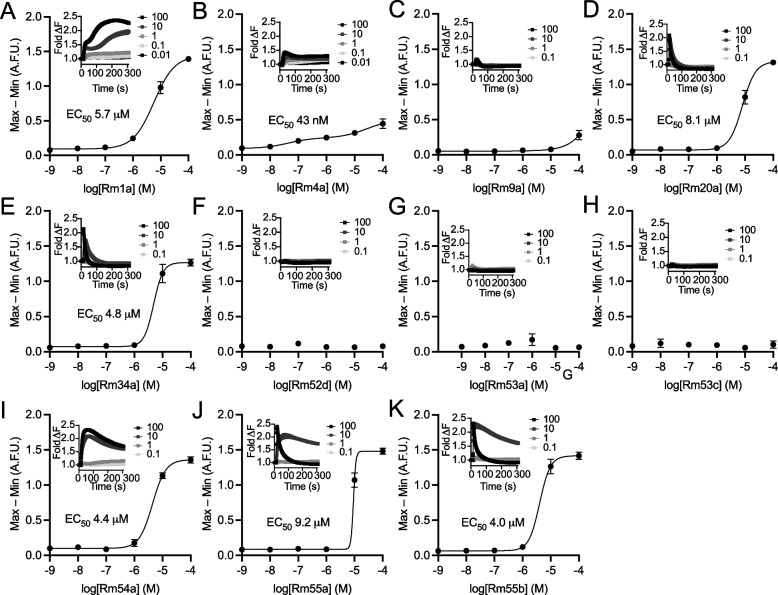
Venom peptides are pharmacologically diverse. Effect of venom peptides on F11 cells: **A** Rm1a, **B** Rm4a, **C** Rm9a, **D** Rm20a, **E** Rm34a, **F** Rm52d, **G** Rm53a/b, **H** Rm53c, **I** Rm54a, **J** Rm55a, and **K** Rm55b. Values are max – min (*n* = 3), and error bars represent SEM. Curves were fitted using a four-parameter Hill equation (variable slope) in Graphpad Prism v8. Inset are the corresponding fluorescence-time traces; error bars show SEM (*n* = 3)

We next sought to identify the venom component/s responsible for the painful effects of envenomation i.e., those which play a putative role in defense. We began by assessing the capacity of each of the 11 peptides to activate mammalian neuronal cells, defined as an increase in intracellular Ca^2+^ concentration ([Ca^2+^]_*i*_) in the F11 (mouse neuroblastoma × rat dorsal root ganglion (DRG) neuron hybrid) cell line (Fig. 5). Of the 11 peptides tested, 7 caused an increase in [Ca^2+^]_*i*_. We then investigated the capacity of these seven peptides to induce nocifensive behavior (licking, biting, and shaking of the injected paw) in mice following intraplantar injection (Fig. 4C). At a dose of 200 pmol, only one of the peptides, Rm4a, produced nocifensive behavior. Its effect began immediately, lasted the entire course of observation, and peaked near maximal (i.e., constant nocifensive behavior) at ~30 min. This characteristic gradual increase in nocifensive behavior over several minutes resembles the reaction to *R. metallica* envenomation in humans. Rm4a was also detected in only half of the individually examined worker venoms, which is consistent with the variable level of pain resulting from stings by this species and suggests that there are likely to be differences in individual worker venom efficacy in defense against vertebrates.

### “Orphan” peptide toxins are pharmacologically diverse

Rm1a and Rm4a appear to play distinct predatory and defensive roles, respectively, and along with closely related peptides of “clade 2” account for almost half of the total venom peptide expression in our pooled individuals of *R. metallica* (44% of total TPM). In contrast, the function(s) of the remaining peptides was less clear. Several of the peptides (Rm20a, Rm34a, Rm54a, Rm55a, and Rm55b) were cytotoxic to HAP cells (Fig. 4D) and our experiments with F11 cells suggested that these peptides had lytic/pore-forming activity, at least at high concentrations (Fig. 5). Further experiments in a range of different cell lines and bacterial strains, demonstrated that the cytolytic properties of these peptides differed (results are summarized in Additional file 4: Fig. S2 and Additional file 5: Table S2). For example, Rm34a caused complete or near-complete growth inhibition of all microbial strains (EC_50_ of ≤ 90 nM for *Staphylococcus aureus* and *Acinetobacter baumannii*), while Rm20a caused complete or near-complete growth inhibition of all microbial strains (EC_50_ of ≤ 100 nM for *A. baumannii*), except *Pseudomonas aeruginosa* and *Candida albicans*. Both Rm54a and Rm55a, caused complete inhibition of *S. aureus* (EC_50_ of 11 μM and ≤ 140 nM, respectively), while not affecting any of the other strains examined. Of the peptides that showed strong antimicrobial activity, all also showed partial toxicity on HEK293T cells and/or human red blood cells. However, at 100 μM, Rm54a was cytotoxic against HEK293T cells but not the melanoma cell line MM96L. Rm9a, Rm52d, Rm53a/b, and Rm53c showed no inhibitory activity on the panel of microbes examined, but 100 μM Rm9a was cytotoxic to both HEK293T and MM96L cells. Thus, while the function(s) of these aculeatoxin peptides remain unknown, they possess distinct pharmacological activities.

### Intra-colony venom variation is genetic and at least partly allelic

**Fig. 6:**
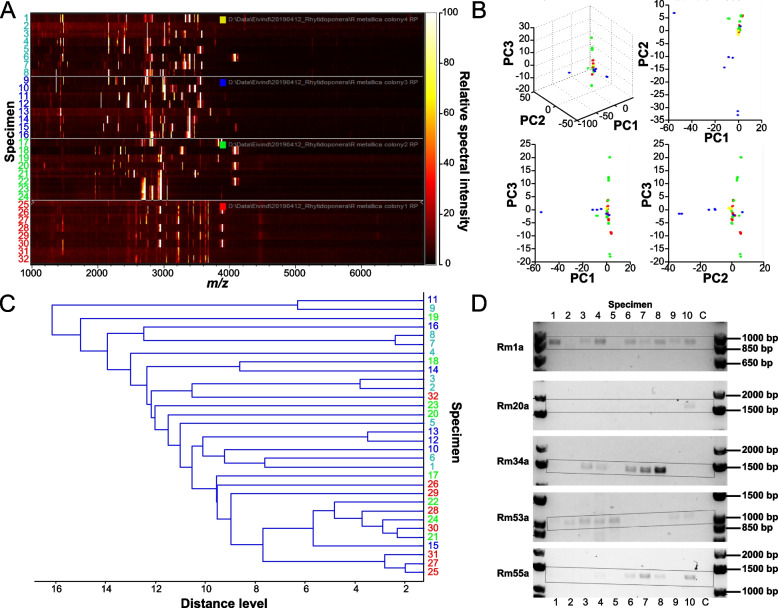
Intra-colony toxin diversity in *R. metallica* is at least partly due to toxin allelic variation. **A** Gel view of MALDI MS spectra collected in positive reflectron mode in three technical replicates from venoms of eight individual workers across four different colonies of *R. metallica*, showing both high intra- and inter-colony variation in venom composition. **B** Principal component analysis (PCA) and **C** clustering analysis of MALDI MS spectra of individual workers from different colonies revealed neither colony-specific clusters nor across-colony clusters suggestive of chemically distinct worker castes. Datapoints in the PCA plots and numbered specimens in the clustering analysis are colored according to the colony label in **A**. **D** PCR products from genomic DNA using toxin-specific primers show that the intra-colony genetic variation is at least in part due to toxin allelic variants. Genomic was extracted from individual ants belonging to the same colony as those used for our transcriptome and main proteomic data. DNA standards and their corresponding length (bp) are shown in the left- and right-most lanes, individual ants are labeled 1–10, while C represents negative control. Full gel images are shown in Additional file 4: Fig. S4

We next examined whether the observed variation in venom composition could be due to phenotypic plasticity that could reflect morphologically similar but chemically distinct worker castes, which have been reported in other ant species [11, 16]. We analyzed the peptide toxin compositions of venoms from individual workers belonging to four nearby colonies using MALDI-MS and compared these using principal component analyses (PCA) and main spectrum profile (MSP) classification analyses (Fig. 5). Our results revealed the same compositional variation between the workers’ venoms in all colonies and that intra-colony variation generally exceeded the differences observed between colonies. We also found no evidence of phenotypic clusters that would suggest the presence of chemical castes (Fig. 6B, C), suggesting that the high colony variance in *R. metallica* aculeatoxin is genetic.

Although the results from our proteotranscriptomic and phylogenetic analyses point to substantial genetic intra-colony variation in *R. metallica*, they do not distinguish between genetic variation contained in gene regulatory elements (i.e., toxin expression levels) and toxin-encoding genes (i.e., toxin alleles). We therefore screened genomic DNA extracted from 10 workers from the same colony for the presence of five aculeatoxins using toxin-specific primers. These primers amplified the genomic region spanning the propeptide and mature toxin, which based on the gene structures of other ant aculeatoxins include at least one intronic region (Additional file 4: Fig. S3). This approach showed that there is substantial variation among individual ants (Fig. 6D, Additional file 4: Fig. S4): not one of the five aculeatoxins was found in all ten specimens, one toxin (Rm1a) was found in eight ants, one toxin was found in seven ants (Rm53a), two toxins were found in five ants (Rm34a and Rm55a), while one toxin was found in only one ant (Rm20a). It should also be noted that although the primers designed to amplify Rm53a appeared to have only moderate specificity—with weak bands indicating additional PCR products—these were only found in specimens with Rm53a. These findings demonstrate that toxin allelic variants provide a substantial contribution to the observed genetic intra-colony variation.

### *R. metallica* aculeatoxins evolve under strong selection

**Fig. 7:**
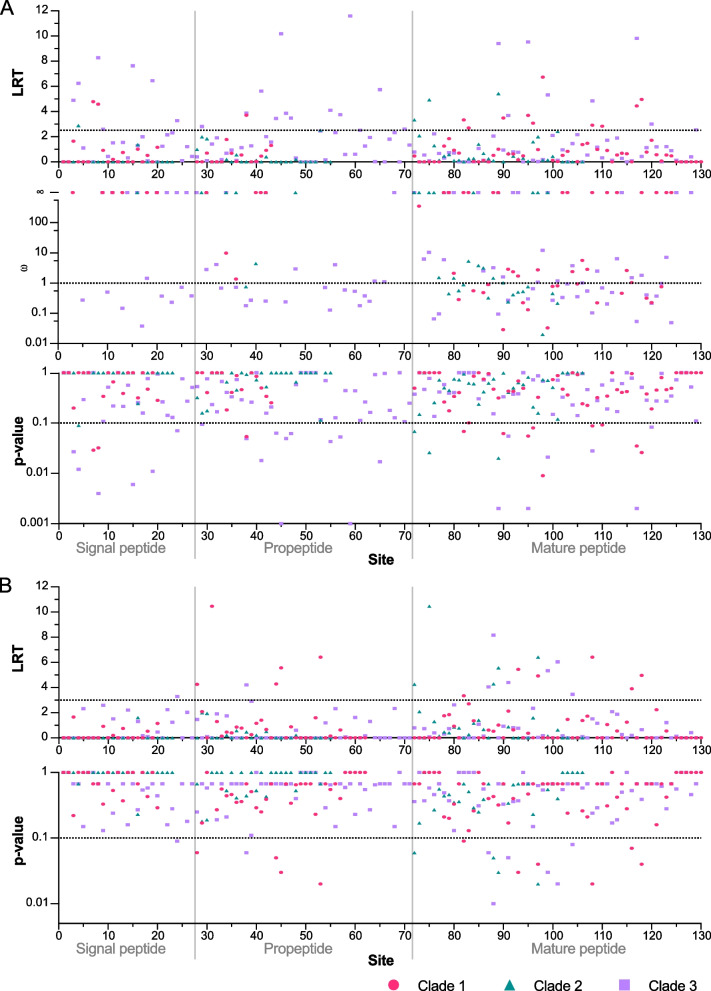
Site-specific selection on *R. metallica* aculeatoxin genes. Log ratio test scores, omega values, and p-values obtained by **A** fixed effects likelihood (FEL) and **B** mixed effects model of evolution (MEME) tests, partitioned according to signal-, pro-, and mature peptide domains as indicated by vertical solid lines. Dotted horizontal lines indicate either significance thresholds (FEL and MEME) or positive (> 1) or negative (< 1) selection (FEL). For plotted values see Additional file 6: Table S3. For alignments, see Additional file 3

Given the structural and functional diversity contained within each of the three main aculeatoxins clades, we next examined whether this diversity could be due to diversifying (positive) selection, which is commonly assumed to be the predominant driver of toxin diversity in animal venoms [17] but at the same time may be expected to reduce population genetic variance. We used *f*ixed *e*ffects *l*ikelihood (FEL) [18] and *m*ixed *e*ffects *m*odel of *e*volution (MEME) [19] tests to look for evidence of pervasive selection and episodic positive selection, respectively (Fig. 7, Additional file 6: Table S3). Both analyses uncovered evidence that the mature domains in each of the three clades have evolved by strong diversifying selection, with FEL/MEME detecting 3/6, 3/5, and 1/6 residues as being under significant positive selection for clades 1, 2, and 3, respectively. These numbers translate to about 20% of the sites in the mature aculeatoxins being under significant positive selection. We also detected several sites under significant negative selection in the mature peptide regions of clades 1 and 3 (FEL; 4 and 6 residues, respectively), although in the case of clade 1 two of these sites correspond to the conserved cysteine residues (boxed, top panel of Fig. 2). In comparison, we identified in the signal peptide domains only 1 site under positive selection (in clade 3) and 2, 1, and 5 sites under significant negative selection in clades 1, 2, and 3, respectively. FEL identified 1 and 10 sites under significant negative selection in the propeptide regions of clades 1 and 3, respectively, while MEME found significant evidence for 5 and 1 sites evolving under episodic positive selection in the same respective clades. These results suggest that despite their high genetic variance, *R. metallica* toxins evolve under strong selection: while the prepropeptide domains involved in transport and processing of the toxins are generally conserved by negative selection, strong positive selection is an important driver of the diversification of the mature aculeatoxins found in *R. metallica* venom.

## Discussion

The toxin diversity contained within the colony-wide venom of *R. metallica* is striking, with more than five times the number of unique toxins compared to some previously described ant venoms [9, 10, 12]. Equally striking, however, is the variation in venom composition between workers that together contribute to this colony-wide toxin diversity. Although it remains to be determined whether this compositional diversity is predominantly due to genetic variation in regulatory (i.e., toxin gene expression) or structural (i.e., toxin alleles) elements, the range of pharmacological properties we observed among the representative peptide toxins show that it translates to a high functional diversity in the colony-wide venom. It has previously been proposed that aculeatoxins may exert their activities predominantly through interactions with cell membranes [9]. However, the intriguing differences in cell selectivity exhibited by these peptides combined with their apparent recent radiation under strong positive selection suggest they may have evolved to target a wide range of different tissue types in prey, predators, or both.

An apparent contrast to the high genetic variation observed in *R. metallica* venom is the findings that the aculeatoxins therein evolve under strong selection, which is often considered a force that reduces population genetic variance. However, there are several scenarios where selection may also increase genetic variation. For example, local selection combined with migration or selection towards temporally moving optima could result in large differences both within and between colonies through spatially or temporally segregated selection for different venom phenotypes. Alternatively, a form of balancing selection that maintains functionally distinct venom phenotypes could also result in the maintenance of different genetic variants that contribute to these phenotypes. Although our results do not allow us to distinguish between these three scenarios, they all suggest that the high genetic variation in the venom of *R. metallica* is the result of adaptation to local ecological factors.

While the evolution of a diverse pharmacological repertoire in *R. metallica* venom can be explained by either of the above evolutionary scenarios, the spatially or temporally varying directional selection scenarios do not explain the maintenance of high genetic variation within the colony, i.e., how the eusocial structure of *R. metallica* colonies is maintained despite the extremely high genetic variance they harbor [6]. This high genetic variance is problematic because high relatedness is a prerequisite for inclusive fitness, which is the basis for kin selection—a key force in maintaining eusociality. However, selection can also act to maintain eusociality in groups with low kinship provided that (i) these groups form well-defined units that selection can act on and (ii) that a population contains multiple such groups, referred to as discrete trait-groups by D. S. Wilson [20]. Moreover, kin selection and group selection are neither necessarily opposing nor mutually exclusive phenomena and may be at work at different levels of biological organization [21, 22]. This is may be the case in *R. metallica*, where colonies consist of well-defined groups of highly related individuals (gamergates and their offspring, subject to kin selection) that have high among-group genetic variance (offspring from different gamergates, comprising discrete trait-groups within colonies that are subject to group balancing selection).

The presence of discrete trait-groups that are comprised of offspring from different gamergates in colonies of *R. metallica* is supported by observations from other ant species. In the ponerine ant *Neoponera inversa* (prev. *Pachychondyla* [23]), for example, colonies consist of unrelated matrilines that perform different tasks in the colony [24]. Although it remains unknown whether this presumably genetically-based behavioral bias is present in *R. metallica*, *R. metallica* workers are opportunistic foragers that individually hunt a wide range of invertebrate prey [25] and even collect non-animal food items such as seeds [26]. We therefore speculate that differences in the pharmacological properties of their venoms could be correlated with prey and other food source preferences. However, regardless of whether this behavioral bias is present, the opportunistic foraging in *R. metallica* means that the presence of multiple venom trait-groups may, on a colony level, lead to greater success in foraging due to the presence of a more diverse toxin repertoire*.*

The diverse colony-level toxin repertoire in *R. metallica* is also likely to be beneficial for nest defense, where the presence of a greater number of venom phenotypes could translate to a greater ability to effectively repeal more types of vertebrate and invertebrate predators, including other ants. A defensive benefit from increased colony-wide toxin diversity has previously been proposed for the social wasp *Polybia paulista*, where high intra-colony genetic variation is also thought to result in high intra-colony toxin diversity [27]. Although the venom of *R. metallica* is not purely defensive, such as in the case of *P. paulista*, it is likely that colonies are under strong selection from predation, and/or competition from other ants, and that the fitness of each colony therefore is linked to the presence of multiple venom trait-groups.

Taken together, we propose that *R. metallica* colonies are subject to both kin- and group selection but that a form of frequency-dependent balancing group selection on the colony may be the uniting force of eusociality in a genetically diverse colony: Gamergates producing offspring with rare functional venom phenotypes could open new food resources for their colony and offer better defense against more predators or colony foragers, thereby disproportionally contributing to the fitness of the colony. In contrast, colonies containing less genetic diversity are more likely to produce workers with similar, non-complementary venom pharmacologies, increasing the likelihood of resource-limited colony growth and increased predation and competition from other ants. This potentially close link between colony-level toxin diversity and resource exploitation and colony defense suggests that the fitness of a *R. metallica* colony is related to the genetic variation it contains, which could contribute to explaining the ecological success of this species despite the high genetic variation in its eusocial colonies.

## Conclusions

Although the causative evolutionary and population genomic mechanisms for the observed toxin gene variation in *R. metallica* remain to be investigated further, our results nevertheless suggest that the increased intra-colony genetic variance resulting from low kinship can by itself provide a selective advantage. The resulting pharmacological and functional diversity of toxins expressed throughout the colony likely enables *R. metallica* colonies to effectively capture a wider range of prey and defend against a wider range of predators, perhaps explaining why they are among the most widespread and abundant ant species on the Australian sub-continent. Thus, group-level selection on phenotypic diversity in adaptive traits could explain the maintenance of eusociality in low-related species by allowing their colonies to exploit a wider set of natural resources.

## Methods

### Venom collection

Adult workers of *R. metallica* used for transcriptome sequencing and our main venom analyses were collected from a 2-m^2^ area of lawn (and were assumed to be from a single colony) at The University of Queensland, St Lucia, Queensland, Australia. Venom was collected from 100 *R. metallica* workers by holding the ant with a pair of forceps while allowing it to sting through a layer of parafilm covering the opening of a 1.5-mL tube (Supplemental Video 1). Ants aggressively and repeatedly stung the parafilm layer presented, depositing, on most occasions, a small drop of clear colorless venom. Collection was continued until no more venom was deposited. Venom from the stings of multiple ants was collected from the bottom of the parafilm layer by rinsing with 10 μL of ultrapure water. All collected venom was pooled then stored at −20°C until further analysis. The total amount of venom collected from 100 individuals, as estimated from A_280_ measured using a NanoDrop spectrophotometer (Thermo Fisher, Waltham, MA, USA), was 370 μg (in a final volume of 200 μL). To compare the venoms of these ants with those of other nearby colonies of *R. metallica*, we also used the same method to obtain venom from four additional colonies across the UQ St Lucia campus, collecting eight workers from the entrances from each colony.

### Transcriptome sequencing and assembly

Venom apparatuses (venom gland filaments, venom reservoir, and venom duct) were dissected from the ants three days after venom collection (using the same individuals from which venom was collected). Using TRIzol (Life Technologies, Carlsbad, CA, USA), total RNA was extracted from the pooled venom apparatuses of 30 individuals. Complementary DNA library preparation and sequencing was performed by the UQ Institute for Molecular Bioscience Sequencing Facility. A dual-indexed library was constructed with the TruSeq-3 Stranded mRNA Sample Prep Kit (Illumina, San Diego, CA, USA) with oligo (dT) selection and an average insert size of 180 base pairs. Samples were pooled in a batch of 20 samples, and 150-cycle paired-end sequencing was performed on an Illumina NextSeq 500 instrument. Adapter trimming of demultiplexed raw reads was performed using fqtrim v0.9.7 [28], followed by quality trimming and filtering using prinseq-lite v0.20.4 [29]. Error correction was performed using BBnorm tadpole, part of the BBtools package. Trimmed and error-corrected reads were assembled using Trinity v2.4.0 [13] with a k-mer length of 31 and a minimum k-mer coverage of 2. Assembled transcripts were annotated using a BLASTX [30] search (E value setting of 1e^−3^) against the UniRef90 database. Estimates of transcript abundance were performed using the RSEM [31] plugin of Trinity (align_and_estimate_abundance). Using TransDecoder, Transcripts were translated and filtered to open-reading frames (> 50 amino acid residues). This was used as a search database for ProteinPilot.

### Mass spectrometry

A combination of top-down of native and reduced and alkylated venom, and bottom-up proteomics of reduced, alkylated and trypsin-digested venom was used to examine the venom composition of *R. metallica*. Two aliquots of venom (10 μg each) were dried by vacuum centrifugation. Gas phase reduction and alkylation was performed according to the protocol described by Hale et al. [32]. 100 μL of reduction/alkylation reagent (50% (v/v) ammonium carbonate, 48.75% acetonitrile (ACN), 1% 2-iodoethanol, 0.25% triethylphosphine) was added to the lid of each 1.5 mL tube containing dried venom, which was then inverted, closed, and incubated at 37°C for 90 min. One aliquot of reduced and alkylated venom was then digested by incubating with trypsin (20 ng/μL) overnight at 37°C, according to the manufacturer’s instructions (Sigma-Aldrich, St. Louis, MO, USA).

Three venom samples (10 μg each)—native venom, reduced and alkylated venom, and reduced, alkylated and trypsin-digested venom—were analyzed by LC-MS/MS. Samples were separated on a Nexera uHPLC (Shimadzu, Kyoto, Japan) with a Zorbax stable-bond C18 column (2.1 × 100 mm; particle size, 1.8 μm; pore size, 300 Å; Agilent, Santa Clara, CA, USA), using a flow rate of 180 μL/min and a gradient of 1 to 40% solvent B (90% ACN and 0.1% formic acid [FA]) in 0.1% FA over 25 min, 40 to 80% solvent B over 4 min, and analyzed on an AB Sciex 5600 TripleTOF (SCIEX, Framingham, MA, USA; operated with Analyst TTF v1.8) mass spectrometer equipped with a Turbo-V source heated to 550°C. MS survey scans were acquired at 300 to 1800 mass/charge ratio (m/z) over 250 ms, and the 20 most intense ions with a charge of +2 to +5 and an intensity of at least 120 counts were selected for MS/MS. The unit mass precursor ion inclusion window mass within 0.7 Da and isotopes within 2 Da were excluded from MS/MS, with scans acquired at 80 to 1400 m/z over 100 ms and optimized for high resolution. Using ProteinPilot v5.0 (SCIEX), MS/MS spectra were searched against the translated venom apparatus transcriptome.

Transcripts identified as encoding venom components in the first ProteinPilot search were used to generate a BLAST database, which, in an effort to identify all venom component homologs in the assembled transcriptome, was aligned using BLASTn (e value 1e^−6^) back to the complete transcriptome. Transcripts encoding venom components and identified homologs were then manually examined using the Map-to-Reference tool of Geneious v10.2.6 [33], where numerous “masked” homologs were extricated from assembled transcripts and erroneous transcripts discarded. These were then reincorporated back into the complete transcriptome, estimation of transcript abundance repeated, and a second, final ProteinPilot search performed. Peptides identified by ProteinPilot were validated by comparison of experimentally derived MS/MS peaks against a theoretical peak list generated using MS-Product in ProteinProspector v5.22.1 (http://prospector.ucsf.edu/prospector/cgi-bin/msform.cgi?form=msproduct).

We also examined the composition of *R. metallica* venom using MALDI MS. 1 μL of diluted venom was mixed with 2 μL diluted a-cyano-4-hydroxycinnamic acid (CHCA) solution (stored as an acetone-saturated solution and diluted 1 in 10 with a solution of ethanol:acetone:0.1% TFA (6:3:1) as a working solution) and spotted onto a polished steel target. MALDI spectra were acquired using a Bruker Autoflex Speed MALDI time-of-flight (TOF)/TOF system (Bruker Daltonics Inc, MA, USA; operated with Flex Control v3.4) in linear reflectron mode at 2000 Hz, with a m/z range of 1,000–7,000. Ten spectra of 500 shots each were saved. The group of 10 spectra were loaded into ClinProt Tools v3.0 (Bruker) as a separate group and observed as a gel view, with the averaged spectrum for the entire dataset produced after recalibration of the entire loaded sample cohort. This dataset was then analyzed by PCA and clustering analysis in ClinProt Tools 3.0 using default settings.

### Gel electrophoresis and in-gel digestion

*R. metallica* venom (10 μg) was denatured (70°C, 10 min) in sample buffer and separated on 4-12% Bis-Tris Plus gels (150 V, 22 min; Life Technologies, CA). Gels were stained with Coomassie Brilliant Blue R-250. Four protein bands > 20 kDa were excised and reduced and alkylated, in gel, according to the protocol described by Hale et al. [32]. Excised bands were destained for 1 h with 50% ACN in 40 mM ammonium bicarbonate, incubated for 90 min at 37°C in reduction/alkylation reagent (50% (v/v) ammonium carbonate, 48.75% ACN, 1% 2-iodoethanol, 0.25% triethylphosphine), rinsed with water then dehydrated with 100% ACN. Trypsin (20 ng/L), prepared according to the manufacturer’s instructions (Sigma-Aldrich, St. Louis, MO), was added to the gel pieces and then incubated for 16 h at 37°C. Reactions were quenched with FA (1% final concentration) and the samples were analyzed by LC-MS/MS as described above.

### Peptide synthesis

Peptides were produced using Fmoc solid-phase peptide synthesis at 0.1 mmol scale. Protecting groups used were Lys/Trp/His(Boc), Ser/Thr/Tyr(tBu), Asp/Glu(OtBu), Asn/Gln/Cys/His(Trt), and Arg(Pbf). Rm1a, Rm4a, Rm20a, Rm52d, Rm53a, Rm54a and Rm55a/b were assembled on Rink-amide ProTide resin (CEM, Matthews, NC) to produce a C-terminal amide, whereas Rm9a and Rm34a were assembled on preloaded Wang-polystyrene resin (ChemImpex, Wood Dale, IL), Leu- and Asn, respectively to achieve an acid C-terminal. For Rm52d and Rm53a, amino acids were coupled manually for 10 min using 4 eq of 0.5 M O-(1H-6-chlorobenzotriazole-1-yl)-1,1,3,3-tetramethyluronium hexafluorophosphate and 8 eq of N,N-diisopropylethylamine, and Fmoc was removed with 20% piperidine (2 × 5 min). Rm1a, Rm4a, Rm9a, Rm20a, Rm34a, Rm53c, Rm54a and Rm55a/b were assembled on a CEM Liberty Prime HT24 microwave synthesizer (CEM Corp, Matthews, NC, USA) using N,N′-diisopropylcarbodiimide/oxyma and Fmoc groups were removed with 20% pyrrolidine, as per manufacturers protocols.

Peptides were released from resin by treatment with 95% TFA/2.5% water/2.5% triisopropylsilane for 50 min at 40 °C on a CEM Razor (CEM, Matthews, NC, USA). Rm54a and Rm53c were cleaved at room temperature while stirring for 2 h using the same cleavage cocktail. Peptides were precipitated with 15 mL ice-cold ether, extracted in A/B 50/50 (A: 0.05% TFA, B: 90% ACN, 0.045% TFA) and lyophilized prior to purification. Due to the hydrophobic nature of the peptides, both ether- and aqueous phases were saved after cleavage. Following lyophilization, Rm1a, Rm4a, Rm53c Rm54a, and Rm55b were not found in the aqueous phase and their respective ether-phases were evaporated and dissolved in A/B for purification. Peptides were purified on a Shimadzu Prominence LC-20AT RP-HPLC system equipped with a SPD-20AV UV detector and a FRC-10A fraction collector using a Agilent C18 column (30 × 250 mm; particle size, 5 μm; pore size, 100 Å; Agilent Technologies, CA, USA) at 8 mL/min. Gradients used were 10–70% B over 60 min (Rm34a, Rm52d, and Rm53a), 20–80% B over 60 min (Rm9a and Rm20a) and 40–90% B over 50 min (Rm1a, Rm4a, Rm53c Rm54a, and Rm55a/b). Fractions of interest were lyophilized and purity assessed using ESI MS and analytical RP-HPLC. Rm9a was oxidized at 0.1 mg/mL in 1 M NH_4_OAc at pH 8.0 over night at room temperature and purified and analyzed as described above.

### Insect incapacitation assays

House crickets (*A. domesticus*; Pisces Live Food, QLD, Australia) (average mass 50 mg), were injected intra-abdominally with 40 nmol/g of each synthetic peptide (in 2 μL water). Crickets were observed for 30 min following injection and compared with negative control crickets injected with 2 μL water. Lethality was assessed at 24 h. For Rm1a, which showed an effect at the highest dose tested (40 nmol/g), a dose-response experiment was performed. To calculate dose-response curves, a four-parameter Hill equation with variable Hill slope was fitted to the data (GraphPad Prism 8.02). All data are expressed as the mean ± standard error of the mean (SEM) and are representative of at least three independent data points. The percentage of crickets incapacitated by a dose of 40 nmol/g, was compared with negative control (water injection) by one-way ANOVA Dunnett’s multiple comparisons test (GraphPad Prism8).

Female fruit flies (*D. melanogaster* strain Canton-S) were injected four to six days post-emergence. Female flies were used because their larger size makes injection easier. Groups of eight female flies were assayed in triplicate for each dose tested. Injection needles were formed from glass capillary tubes (#3-000-203-G/X, Drummond, Birmingham, AL, USA) using a micropipette puller (Sutter Instrument Co. Model P-97), with the fine tip manually trimmed afterwards using scissors. Needles then were filled with mineral oil, connected to a Nanoliter 2000 microinjector (Kanetec, Bensenville, IL USA) equipped with a foot pedal, and ~1 μL of peptide solution or water aspirated. Each group of flies were immobilized by cooling for approximately 2 min on ice, and then decanted on the top of the ‘injection stage’, a petri dish filled with ice. Under a dissecting microscope, each fly was carefully impaled on the lateral thorax behind the wing, and 50.6 nL of venom peptide or water was injected. After injection, each group of flies were returned to their 5 mL plastic tube at room temperature and their behavior observed. Climbing behavior was assayed at 5 min by tapping the tube five times on the table to dislodge flies to the bottom and counting the number that failed to climb from the bottom of the tube after 5 s.

### Calcium imaging of F11 cells

F11 (mouse neuroblastoma × rat dorsal root ganglion (DRG) neuron hybrid) cells were cultured as previously described [34]. Cells were maintained on Ham’s F12 media supplemented with 10 % fetal bovine serum, 100 μM hypoxanthine, 0.4 μM aminopterin, and 16 μM thymidine (Hybri-MaxTM, Sigma Aldrich). 384-well imaging plates (Corning, Lowell, MA, USA) were seeded 48 h prior to imaging resulting in 90 – 95% confluence at imaging. Cells were incubated for 30 min with Calcium 4 assay component A, as per the manufacturer’s instructions (Molecular Devices, Sunnyvale, CA) in physiological salt solution (PSS; composition in mM: 140 NaCl, 11.5 D-glucose, 5.9 KCl, 1.4 MgCl_2_, 1.2 NaH_2_PO_4_, 5 NaHCO_3_, 1.8 CaCl_2_, 10 HEPES) at 37°C. Ca^2+^ responses were measured using a FLIPR^TETRA^ fluorescent plate reader equipped with a CCD camera (Ex: 470–490 nm, Em: 515–575 nM) (Molecular Devices, Sunnyvale, CA). Signals were read every second for 10 s before, and 300 s after, the addition of peptides in PSS supplemented with 0.1% bovine serum albumin. All data represent mean ± SEM of a representative assay in triplicate. Fluorescence changes on peptide addition were compared to addition of negative control solution (0.1% BSA in PSS). The resulting maximum-minimum fluorescence in the 300 s period after peptide addition was recorded as the response. A four-parameter Hill equation (variable slope) was fitted (a two-site fit was used for the Rm4a data) using Graphpad Prism8.

### Pain behavior experiments

Male adult (6 weeks old) C57BL/6J mice were used for behavioral experiments. Each peptide (200 pmol) diluted in saline containing 0.1% bovine serum albumin (BSA) was administered in a volume of 20 μL into the hind paw by shallow intraplantar injection. Negative control animals were injected with saline containing 0.1% BSA. Following injection, spontaneous nocifensive behavior events were counted from video recordings by a blinded observer. Nocifensive behavior events were summed over 30 min and compared with negative control (saline injection) by one-way ANOVA Dunnett’s multiple comparisons test (GraphPad Prism8).

### Antimicrobial and cytotoxicity assays

Antimicrobial and cytotoxicity assays were performed by CO-ADD (The Community for Antimicrobial Drug Discovery, Institute for Molecular Bioscience, The University of Queensland). Growth inhibition of each bacterial strain was determined by measuring absorbance at 600 nm, whereas growth inhibition of *C. albicans* was determined by measuring absorbance at 530 nm, and growth inhibition of *C. neoformans* was determined by measuring the difference in absorbance between 600 and 570 nm, after the addition of resazurin (0.001% final concentration) and incubation at 35°C for an additional 2 h. Growth inhibition of HEK293T cells was determined by measuring fluorescence (excitation 530/10 nm, emission 590/10 nm) after the addition of resazurin (25 μg/mL final concentration) and incubation at 37°C and 5% CO_2_, for an additional 3 h. Percentage growth inhibition was calculated using negative controls (media only) and positive controls (no peptide). For the hemolysis assays, human whole blood was washed three times with 3 volumes of 0.9% NaCl and then resuspended in 0.9% NaCl to a concentration of 0.5 × 10^8^ cells/mL. Cells were incubated for 1 h at 37°C with or without the peptide. After incubation, plates were centrifuged at 1000 *g* for 10 min to pellet cells and debris and hemolysis determined by measuring the supernatant absorbance at 405 nm. Minimum inhibitory concentration (MIC), CC_50_ (concentration at 50% cytotoxicity) and HC_50_ (concentration at 50% hemolytic activity) values were calculated by curve fitting the inhibition values versus log(concentration) using a sigmoidal dose-response function (variable slope), in Pipeline Pilot’s dose-response component.

In addition to the cytotoxicity assays performed by CO-ADD, we screened the synthetic peptides for toxicity in cell viability assays using HAP1, MM96L, and HEK293T cells. peptides were reconstituted in 100% DMSO then diluted in cell media (HAP1: IMDM (Sigma Aldrich) with 10% FBS and 1% Penicillin/Streptomycin (P/S); MM96L: RPMI (Gibco) with 10% FBS, 1% P/S and 1% GlutaMAX (Gibco); HEK293T: DMEM high glucose (Gibco), with 10% FBS and 1% P/S. Trypsinized cells (35 × 104) were seeded in each well of a 96-well plate. 0.01 to 1000 μM peptide was added to each well after 24 h, and the cells incubated for an additional 74 h. After incubation, the medium was aspirated from each well and 150 μL of fresh medium containing a 0.002% solution of resazurin (Sigma-Aldrich) was added to the wells and incubated for 4 h at 37 °C. The absorbance was measured at 570 nm using a microplate spectrophotometer (FLUOstar Omega, BMG Labtech, Germany). Percentage cell viability at concentrations of 10 and 100 μM peptide were compared with negative control by one-way ANOVA Dunnett’s multiple comparisons test (GraphPad Prism8).

### Molecular evolution analyses

*R. metallica* aculeatoxin peptide sequences were BLAST searched against UniProtKB and aligned with the resulting homologs as well as previously identified aculeatoxins from ants using an iterative refinement method incorporating local pairwise alignment information (L-INS-i) in MAFFT v7.304b [35]. We then used a molecular phylogenetic approach to examine the evolutionary history of the identified aculeatoxin peptides. The most appropriate evolutionary model was identified using ModelFinder [36] and reconstructed molecular phylogenies by maximum likelihood with IQ-TREE multicore v2.0.6 [37], estimating branch support values by ultrafast bootstrap using 10,000 replicates [38].

Due to the extreme divergence between the mature aculeatoxin peptide domains compared to the signal and propeptide domains, we also searched for evidence of recombination within each of the three main clades of *R. metallica* aculeatoxins. We used a sequence threader to thread nucleotide sequences to amino acid sequence alignments generated for each clade using L-INS-i in MAFFT v7.304b (Fig. 2) [39], and then looked for evidence of up to two recombination break points (between each domain type) using the Genetic Algorithm for Recombination Detection (GARD) [40] implemented in Datamonkey [41]. Because this analysis revealed evidence of breakpoints between the pro- and mature peptide domains in all clades, with clades 2 and 3 possibly representing true recombination breakpoints as opposed to differences in evolutionary rates (Additional file 4: Table S4), we performed two phylogenetic analyses based on alignments of either the full coding regions or only the signal and N-terminal propeptide domains. These analyses returned near-identical topologies (Additional file 4: Fig. S5), suggesting any recombination events occur within clades and that the overall topology generated by our phylogenetic analyses is robust. Nevertheless, we used separate nucleotide alignments for each domain type—signal peptide, N-terminal propeptide, and mature peptide domains—to search for evidence of selection in clades 1–3. FEL tests [18], assuming synonymous rate variation, were used to look for evidence of site-specific pervasive positive and negative selection, and MEME tests [19] were used to look for signs of site-specific episodic positive selection.

### DNA extraction and amplification via PCR

Ten adult workers of *R. metallica* were collected from the same 2 m^2^ area of lawn from which ants for venom collection and transcriptomic studies were collected. Each individual was euthanized by cooling in a freezer, the cuticle was removed via dissection in PBS, and the remaining soft tissue was used for TRIzol (Life Technologies, Carlsbad, CA, USA) DNA extraction. Primer-BLAST [42] was used to design target-specific primers (Additional file 4: Table S5) for the amplification of Rm1a, Rm20a, Rm34a, Rm53a, Rm55a and DPP-4. Primers were synthesized by Sigma-Aldrich (Sigma-Aldrich, St. Louis, MO, USA). For each Polymerase Chain Reaction (PCR) we used ca. 500 ng ant DNA as estimated using a NanoDrop spectrophotometer (Thermo Fisher, Waltham, MA, USA), 0.4 μM primer (forward and reverse), 2 μM dNTP mix (Thermo Fisher, Waltham, MA, USA), 2 μM MgCl_2_ (Fisher Biotec Australia, Wembley, WA, AUS), 1X reaction buffer (Fisher Biotec Australia, Wembley, WA, AUS), and 1 unit TAQ polymerase (Fisher Biotec Australia, Wembley, WA, AUS). PCR was performed using the following cycling conditions: 94°C for 5 min, followed by 30 cycles of 94°C for 2 min, 57–63°C for 2 min, 74°C for 1–2 min, and a final elongation step at 74°C for 10 min. PCR products were separated and visualized on 1% agarose gels with SYBR-safe DNA stain ((Thermo Fisher, Waltham, MA, USA)). Each well was loaded with 10 μL PCR product, either 2 μL 6X loading dye (Thermo Fisher, Waltham, MA, USA)) or 2 μL 1Kb Plus DNA Ladder (Thermo Fisher, Waltham, MA, USA)), and 2 μL loading dye, and separated at 110 V for 55 min.

### Statistics

All data were plotted and analyzed using Graphpad Prism (v9.0.0). Data were fitted to equations as indicated. Statistical significance was defined as *P* < 0.05 using tests as indicated. All data are presented as means ± SEM.

## Supplementary Information

**Additional file 1: Video S1.** Venom extraction from *R. metallica.* Naturally secreted venom was obtained by holding each ant and allowing it to sting through a thin parafilm membrane covering a 1.5 mL Eppendorf tube. Venom is visible as droplets on the underside of the parafilm membrane.

**Additional file 2: Table S1.** Peptides and proteins identified in the pooled venom of R. metallica. Peptide and protein names and corresponding encoding contig id are provided for each, along with the best BLAST hit against UniProtKB, evidence supporting their presence in the venom, mature molecular weight, expression level (TPM), and the sequence of the mature domain and full prepropeptide. Complete (1) evidence includes identification of the complete mature peptide sequence from either the bottom-up or top-down proteomic experiments, while “partial” includes only partial identification from the bottom-up experiment.

**Additional file 3.** Zip file containing sequence alignments used for reconstruction of aculeatoxins phylogeny and GARD, MEME, and FEL tests.

**Additional file 4: Figure S1.** Activity of Rm1a and Rm4a. **Figure S2.** Cytotoxicity of *R. metallica* aculeatoxin peptides. **Figure S3.** Gene structures of some ant aculeatoxins. **Figure S4.** Presence of aculeatoxin alleles among workers from the same *R. metallica* colony. **Figure S5.** Phylogeny of *R. metallica* aculeatoxins estimated from full-length or only signal- and propeptide domains. **Table S4.** Recombination analysis of *R. metallica* clades 1–3*.***Table S5.** Primers used to amplify aculeatoxins from *R. metallica* genomic DNA.

**Additional file 5: Table S2.** Antimicrobial and cytotoxic activity of *R. metallica* aculeatoxins.

**Additional file 6: Table S3.** Results from fixed effects likelihood (FEL) and mixed effects model of evolution (MEME) tests for selection on R. metallica aculeatoxin genes.

## Data Availability

Raw sequencing data have been deposited in the NCBI sequence read archive with dataset identifier SRR13051311 [43]. Prepropeptide sequences of venom peptides have been deposited with GenBank, under accessions: MW317022 - MW317128. Mass spectrometry data and database search results for proteomic experiments have been deposited to the ProteomeXchange Consortium via the PRIDE [44] partner repository with the dataset identifier PXD037863 [45].
