# Supplementary material for: Intra-colony venom diversity contributes to maintaining eusociality in a cooperatively breeding ant
Source: BMC Biol. 2023 Jan 8;21:5. doi: 10.1186/s12915-022-01507-9 (PMC9827630; doi:10.1186/s12915-022-01507-9)
Supplement: Supplementary file 2 — Additional file 2: Table S1. Peptides and proteins identified in the pooled venom of R. metallica. Peptide and protein names and corresponding encoding contig id are provided for each, along with the best BLAST hit against UniProtKB, evidence supporting their presence in the venom, mature molecular weight, expression level (TPM), and the sequence of the mature domain and full prepropeptide. Complete (1) evidence includes identification of the complete mature peptide sequence from either the bottom-up or top-down proteomic experiments, while “partial” includes only partial identification from the bottom-up experiment. [file 12915_2022_1507_MOESM2_ESM.pdf]

| Name | Contig id. | Best BLAST hit (UniProtKB) | Evidence (1 c |
|---|---|---|---|
| ECTX1-Rm1a | TRINITY_DN17344_c5_g3_i1 | noblastxhit | 1 |
| ECTX1-Rm1b | TRINITY_DN17258_c4_g1_i1 | noblastxhit | 1 |
| ECTX1-Rm1c | TRINITY_DN17282_c0_g1_i2 | noblastxhit | 1 |
| ECTX1-Rm2a | TRINITY_DN17358_c2_g1_i1 | noblastxhit | 1 |
| ECTX1-Rm3a | TRINITY_DN17299_c0_g1_i1 | noblastxhit | 0 |
| ECTX1-Rm3b | TRINITY_DN17299_c0_g1_i101 | noblastxhit | 1 |
| ECTX1-Rm3c | TRINITY_DN17299_c0_g1_i102 | noblastxhit | 0 |
| ECTX1-Rm3d | TRINITY_DN17299_c0_g1_i103 | noblastxhit | 1 |
| ECTX1-Rm3e | TRINITY_DN17299_c0_g1_i104 | noblastxhit | 1 |
| ECTX1-Rm4a | TRINITY_DN17299_c0_g1_i105 | noblastxhit | 1 |
| ECTX1-Rm4b | TRINITY_DN17299_c0_g1_i106 | noblastxhit | 0 |
| ECTX1-Rm5a | TRINITY_DN17299_c0_g1_i107 | noblastxhit | 1 |
| ECTX1-Rm5b | TRINITY_DN17299_c0_g1_i108 | noblastxhit | 0 |
| ECTX1-Rm20a | TRINITY_DN17330_c5_g1_i1 | UniRef90_Q07932_Pilosulin | 1 |
| ECTX1-Rm6a | TRINITY_DN17358_c4_g1_i1 | noblastxhit | 1 |
| ECTX1-Rm9a | TRINITY_DN17316_c3_g1_i1 | noblastxhit | 1 |
| ECTX1-Rm38a | TRINITY_DN17357_c16_g1_i1 | noblastxhit | 1 |
| ECTX1-Rm16a | TRINITY_DN15109_c3_g1_i1 | noblastxhit | 1 |
| ECTX1-Rm53a | TRINITY_DN17343_c2_g2_i1 | noblastxhit | 0 |
| ECTX1-Rm6c | TRINITY_DN17346_c1_g1_i1 | noblastxhit | 1 |
| ECTX1-Rm34a | TRINITY_DN17311_c2_g1_i1 | UniRef90_Q68Y23_Pilosulin | 1 |
| ECTX1-Rm37a | TRINITY_DN17353_c3_g1_i1 | UniRef90_Q07932_Pilosulin | 1 |
| ECTX1-Rm39b | TRINITY_DN17352_c0_g1_i1 | UniRef90_Q26464_Pilosulin | 1 |
| ECTX1-Rm6e | TRINITY_DN17344_c5_g1_i1 | noblastxhit | 1 |
| ECTX1-Rm53c | TRINITY_DN17343_c2_g2_i102 | noblastxhit | 0 |
| ECTX1-Rm6d | TRINITY_DN17329_c0_g1_i4 | noblastxhit | 1 |
| ECTX1-Rm55a | TRINITY_DN17336_c1_g1_i102 | UniRef90_Q68Y22_Pilosulin | 1 |
| ECTX1-Rm6f | TRINITY_DN17333_c3_g1_i1 | noblastxhit | 1 |
| ECTX1-Rm53b | TRINITY_DN17343_c2_g2_i101 | noblastxhit | 0 |
| ECTX1-Rm33b | TRINITY_DN17322_c2_g1_i2 | UniRef90_Q07932_Pilosulin | 1 |
| ECTX1-Rm55b | TRINITY_DN17336_c1_g1_i103 | UniRef90_Q68Y22_Pilosulin | 1 |
| ECTX1-Rm41a | TRINITY_DN17328_c1_g1_i4 | UniRef90_Q68Y23_Pilosulin | 1 |
| ECTX1-Rm52d | TRINITY_DN17334_c1_g1_i5 | UniRef90_Q68Y23_Pilosulin | 1 |
| ECTX1-Rm24a | TRINITY_DN17338_c4_g1_i2 | UniRef90_Q26464_Pilosulin | 1 |
| ECTX1-Rm8a | TRINITY_DN17349_c0_g1_i1 | noblastxhit | 2 |
| ECTX1-Rm17a | TRINITY_DN16789_c2_g2_i1 | noblastxhit | 0 |
| ECTX1-Rm14a | TRINITY_DN17257_c0_g1_i3 | noblastxhit | 1 |
| ECTX1-Rm18a | TRINITY_DN24565_c1_g1_i1 | noblastxhit | 2 |
| ECTX1-Rm56b | TRINITY_DN17320_c3_g1_i4 | UniRef90_Q07932_Pilosulin | 1 |
| ECTX1-Rm7a | TRINITY_DN17207_c6_g1_i1 | noblastxhit | 2 |
| ECTX1-Rm39a | TRINITY_DN17323_c1_g1_i1 | UniRef90_Q07932_Pilosulin | 1 |
| ECTX1-Rm13a | TRINITY_DN17209_c0_g1_i1 | noblastxhit | 1 |
| ECTX1-Rm29a | TRINITY_DN17338_c4_g1_i4 | UniRef90_Q26464_Pilosulin | 0 |
| ECTX1-Rm30a | TRINITY_DN17320_c3_g1_i3 | noblastxhit | 1 |
| ECTX1-Rm33a | TRINITY_DN17296_c3_g1_i1 | noblastxhit | 1 |
| ECTX1-Rm31a | TRINITY_DN17306_c3_g1_i1 | UniRef90_Q26464_Pilosulin | 1 |
| ECTX1-Rm45a | TRINITY_DN17341_c1_g1_i2 | UniRef90_Q07932_Pilosulin | 1 |

| | | | |
|---|---|---|---|
| ECTX1-Rm46a | TRINITY_DN17156_c2_g3_i2 | noblastxhit | 1 |
| ECTX1-Rm22a | TRINITY_DN17357_c16_g2_i1 | UniRef90_Q07932_Pilosulin | 1 |
| ECTX1-Rm44a | TRINITY_DN17291_c6_g1_i4 | UniRef90_Q26464%Pilosulir | 2 |
| ECTX1-Rm6b | TRINITY_DN17358_c4_g1_i101 | noblastxhit | 0 |
| ECTX1-Rm11b | TRINITY_DN17319_c1_g1_i201 | noblastxhit | 2 |
| ECTX1-Rm13b | TRINITY_DN17209_c0_g1_i101 | noblastxhit | 1 |
| ECTX1-Rm11c | TRINITY_DN17319_c1_g1_i202 | noblastxhit | 2 |
| ECTX1-Rm32a | TRINITY_DN17291_c6_g1_i1 | noblastxhit | 0 |
| ECTX1-Rm25a | TRINITY_DN17338_c4_g1_i5 | UniRef90_Q26464_Pilosulin | 1 |
| ECTX1-Rm23a | TRINITY_DN17156_c2_g3_i4 | noblastxhit | 0 |
| ECTX1-Rm54a | TRINITY_DN17336_c1_g1_i1 | UniRef90_Q68Y22_Pilosulin | 0 |
| ECTX1-Rm15a | TRINITY_DN17207_c6_g1_i2 | noblastxhit | 2 |
| ECTX1-Rm27a | TRINITY_DN17261_c2_g1_i1 | noblastxhit | 1 |
| ECTX1-Rm44b | TRINITY_DN17291_c6_g1_i401 | noblastxhit | 2 |
| ECTX1-Rm11a | TRINITY_DN17319_c1_g1_i2 | noblastxhit | 2 |
| ECTX1-Rm42a | TRINITY_DN17320_c3_g1_i1 | noblastxhit | 0 |
| ECTX1-Rm36a | TRINITY_DN17274_c0_g1_i6 | UniRef90_Q68Y23_Pilosulin | 1 |
| ECTX1-Rm31b | TRINITY_DN17306_c3_g1_i101 | UniRef90_Q26464_Pilosulin | 1 |
| ECTX1-Rm43a | TRINITY_DN17311_c3_g1_i1 | UniRef90_Q07932_Pilosulin | 0 |
| ECTX1-Rm35a | TRINITY_DN17317_c2_g2_i1 | UniRef90_Q07932_Pilosulin | 0 |
| ECTX1-Rm19a | TRINITY_DN17315_c0_g1_i1 | noblastxhit | 1 |
| ECTX1-Rm52c | TRINITY_DN17334_c1_g1_i502 | UniRef90_Q68Y23_Pilosulin | 0 |
| ECTX1-Rm10a | TRINITY_DN17207_c6_g1_i4 | noblastxhit | 0 |
| ECTX1-Rm52b | TRINITY_DN17334_c1_g1_i501 | UniRef90_Q68Y23_Pilosulin | 0 |
| ECTX1-Rm40a | TRINITY_DN17320_c3_g1_i6 | noblastxhit | 2 |
| ECTX1-Rm12a | TRINITY_DN17309_c3_g1_i3 | noblastxhit | 0 |
| ECTX1-Rm6h | TRINITY_DN17340_c0_g1_i2 | noblastxhit | 0 |
| ECTX1-Rm56a | TRINITY_DN17301_c5_g3_i1 | noblastxhit | 0 |
| ECTX1-Rm48a | TRINITY_DN17320_c3_g1_i5 | noblastxhit | 1 |
| ECTX1-Rm51a | TRINITY_DN17261_c2_g1_i3 | noblastxhit | 0 |
| ECTX1-Rm6g | TRINITY_DN17340_c0_g2_i1 | noblastxhit | 0 |
| ECTX1-Rm26a | TRINITY_DN17230_c2_g5_i2 | noblastxhit | 0 |
| ECTX1-Rm28a | TRINITY_DN17230_c2_g5_i1 | noblastxhit | 0 |
| ECTX1-Rm52a | TRINITY_DN17269_c3_g1_i1 | noblastxhit | 0 |
| ECTX1-Rm21a | TRINITY_DN17200_c5_g1_i1 | noblastxhit | 0 |
| ECTX1-Rm50a | TRINITY_DN17261_c2_g1_i2 | noblastxhit | 0 |
| ECTX1-Rm47a | TRINITY_DN17305_c0_g2_i6 | UniRef90_Q07932_Pilosulin | 0 |
| ECTX1-Rm49a | TRINITY_DN17274_c0_g1_i2 | noblastxhit | 0 |
| ECTX1-Rm57a | TRINITY_DN17098_c0_g2_i1 | noblastxhit | 1 |
| ECTX1-Rm57b | TRINITY_DN17098_c0_g2_i102 | noblastxhit | 1 |
| ECTX1-Rm57c | TRINITY_DN17098_c0_g2_i103 | noblastxhit | 1 |
| ECTX1-Rm58a | TRINITY_DN17273_c4_g1_i2 | noblastxhit | 1 |
| ECTX1-Rm58b | TRINITY_DN17273_c4_g1_i3 | noblastxhit | 1 |
| ECTX1-Rm59a | TRINITY_DN17273_c4_g1_i4 | noblastxhit | 0 |
| ECTX1-Rm59b | TRINITY_DN17273_c4_g1_i402 | noblastxhit | 0 |
| ECTX1-Rm60a | TRINITY_DN7850_c0_g1_i1 | noblastxhit | 1 |
| ECTX1-Rm61a | TRINITY_DN17262_c0_g1_i3 | noblastxhit | 1 |
| ECTX1-Rm61b | TRINITY_DN17259_c0_g1_i2 | noblastxhit | 1 |

| | | | |
|---|---|---|---|
| ECTX1-Rm61c | TRINITY_DN17326_c0_g1_i1 | noblastxhit | 1 |
| ECTX1-Rm62a | TRINITY_DN16464_c2_g2_i1 | UniRef90_UPI000D534F59% | 1 |
| ECTX1-Rm63a | TRINITY_DN21815_c0_g1_i1 | UniRef90_UPI000D534F59% | 1 |
| ECTX1-Rm64a | TRINITY_DN15428_c0_g1_i1 | UniRef90_A0A195ECK0%Un | 2 |
| ECTX2-Rm1a | TRINITY_DN17090_c1_g4_i201 | UniRef90_U3JDX4_Betacellu | 2 |
| ECTX2-Rm1b | TRINITY_DN17090_c1_g4_i2 | UniRef90_U3JDX4_Betacellu | 2 |
| ECTX2-Rm1c | TRINITY_DN17090_c1_g4_i202 | UniRef90_U3JDX4_Betacellu | 2 |
| ECTX2-Rm1d | TRINITY_DN17090_c1_g4_i203 | UniRef90_U3JDX4_Betacellu | 2 |
| ECTX2-Rm1e | TRINITY_DN17090_c1_g4_i204 | UniRef90_U3JDX4_Betacellu | 2 |
| crustacean neurohoi | TRINITY_DN17116_c1_g3_i1 | UniRef90_A0A1B6DZ35%Ur | 2 |
| DPP-4 | TRINITY_DN15828_c0_g1_i1 | UniRef90_UPI000623B729% | 2 |
| Phosphatase | TRINITY_DN17084_c0_g2_i1 | UniRef90_UPI000D537B77% | 2 |
| PLA1 | TRINITY_DN17147_c1_g1_i1 | UniRef90_UPI00063EFA12% | 2 |
| PLA2 | TRINITY_DN17242_c4_g1_i1 | UniRef90_UPI00062367FC% | 2 |
| CAP (11) | | VA3 | 2 |

| Mature MW | TPM |
| --- | --- |
| 2666.53 | 332570 |
| 2701.48 | 23590 |
| 2694.46 | 1234 |
| 3375.84 | 6366 |
| 2527.36 | 1709 |
| 2553.36 | 273 |
| 2547.42 | 2387 |
| 2521.41 | 311 |
| 2420.36 | 4706 |
| 2993.70 | 59069 |
| 2979.65 | 1613 |
| 3217.78 | 5372 |
| 3805.15 | 518 |
| 2533.57 | 46450 |
| 2955.44 | 39106 |
| 3056.48 | 36501 |
| 3230.76 | 29246 |
| 8730.48 | 24305 |
| 1091.57 | 20450 |
| 3007.56 | 19210 |
| 2793.69 | 18512 |
| 4194.13 | 17371 |
| 2165.25 | 16881 |
| 3693.91 | 15407 |
| 1355.72 | 15115 |
| 3399.68 | 14930 |
| 1843.15 | 13413 |
| 3786.83 | 12099 |
| 1091.57 | 10454 |
| 2842.66 | 8084 |
| 2236.40 | 7866 |
| 1875.09 | 6769 |
| 1440.83 | 6171 |
| 3287.81 | 4447 |
| 3203.66 | 4286 |
| 2703.46 | 3645 |
| 3059.85 | 3549 |
| 4225.58 | 3320 |
| 3205.96 | 3175 |
| 4571.47 | 3145 |
| 2147.30 | 3137 |
| 3519.79 | 3077 |
| 1539.01 | 3039 |
| 2363.55 | 2923 |
| 2968.78 | 2733 |
| 3782.14 | 2343 |
| 3130.77 | 2309 |

| | |
|---|---|
| 3565.96 | 2048 |
| 2512.49 | 1960 |
| 2424.42 | 1909 |
| 2978.51 | 1850 |
| 3027.49 | 1711 |
| 3689.95 | 1680 |
| 3059.46 | 1665 |
| 2393.32 | 1652 |
| | 1566 |
| 1225.72 | 1521 |
| 2193.32 | 1209 |
| | 1183 |
| 3690.07 | 1177 |
| 2525.42 | 1104 |
| 3011.53 | 1089 |
| 2100.19 | 1029 |
| 1656.01 | 979 |
| 3800.09 | 905 |
| 3029.01 | 890 |
| 2423.56 | 729 |
| 1539.95 | 625 |
| 1544.90 | 614 |
| 3015.47 | 562 |
| 852.61 | 547 |
| 3326.96 | 530 |
| 3148.66 | 484 |
| 3717.85 | 367 |
| 2390.23 | 347 |
| 2617.52 | 318 |
| 3841.10 | 309 |
| 3702.85 | 295 |
| 1216.70 | 293 |
| 3223.96 | 189 |
| 1530.97 | 111 |
| 1739.94 | 70 |
| 3975.20 | 54 |
| | 45 |
| 2837.60 | 42 |
| 1544.79 | 535 |
| 1429.78 | 535 |
| 1471.79 | 535 |
| 1525.92 | 2385 |
| 1570.01 | 3839 |
| 1391.88 | 6119 |
| 1391.88 | 6119 |
| 1462.65 | 6442 |
| 3121.56 | 18713 |
| 3133.60 | 1237 |

| | |
|---|---|
| 3267.76 | 145 |
| 3167.66 | 129 |
| 2727.31 | 2460 |
| | 612 |
| 7227.30 | 1035 |
| 7227.30 | 1073 |
| 7560.44 | 809 |
| 8861.14 | 262 |
| 8839.13 | 274 |
| 11172.29 | 407 |
| 90kDa | 147 |
| 48kDa | 222 |
| 36kDa | 1042 |
| | 36 |
| 28 kDa | 6102 |

Mature

VAPIVAMAGLGLFAAAVAGLDWLSKKV*
VAPIMAMAGLGLFAAAVAGMDWLAKKV*
VAPIVAMAGLGLFAVAAAGLDWMSKHV*
IAPIVALLAFSLFSSLPFLHYYVTHTTEKP
FLPALPLMLGMMTLPFIHDAATK*
FLPALPLMLGMMTLPFIYDAATK*
ILPVLPLMLGMMTLPFIYDAATK*
VLPLLPLMLGMMTLPFIHDAATK*
VLPLLPLMLGMMTLPFIHDAAK*
FPPLLLLAGLFSLPALQHYIETKWIN*
FPPLLLLAGLFSLPALQHYIETNWIN*
ALPALPLLAFFFTFFPAAQHWLEKKIMA*
ALPALPLLAFFFYLFPGRTALAREKNNGGLKRRS
ALWDTIKKWVAKVLPLVKNALK*
MNWGKLKEMAKKMCPMAKAAMEKACK
SNMQEIMKKLCTDKKMPAWMCKMLGL
FWAAVAKWIIPAALSYAPDVIEWMKNKL*
WNMEEELKKMIPKEIYEKALNKQNELMACVKNVMKI
FWLKGGRGDG*
LNLGKLKEMVKKMCPMAKAAMEKWCK
FIGGLIKFIAKFVPKILPFFQKKN
FWAGFFKTVGTAMLPYMIDKMPEMAKWMQEKIKQI*
AFKDVMQKIGSGLLKALSFI*
IKWSNLKKWLNKDNMKKMCPVAKAAMEKMCK
FWLKAGRGTGYT*
FNWKKWLNKDNMKKMCPVAKAAMEKMCK
FLPFLIPVIASLVSSLK*
INWPNWMTKIFTKDNMKKMCPMAKAAMEKACK
FWLKGGRGDG*
FWGALWRLAKIIPSAIGGLFGGKKKQ
ILPALIPVFASLLPWAIKKF*
WISKVWKGIKMAGKML*
RNVPRWFTPRL*
LNWIKMLTGMGKWVAKKAIGHFMKKKNE
KTKIKEMCANNKIPWKRMCEWVNKRA
RLNLKGIGWKSRGPSFRAGTDPSFG*
LDVKDLLKKLCAKIPDTIPLKKKLCPK
VDWKGFGSKIKRFFKKHKSTIAKTVLKIVTTVLPLVL*
IKISDIIKLAKTVLAALPQNIKDMWKKK*
FNMEEINAKIKEICARKYSNQKISKWIKIACELVKKYI
ALKDVLKNIGSALLKALSFF*
ELNIDKWKEMMKKMCAKMPEPIKKMCPKL
IKWGKLLKTAVGLI*
IKISDIIKKAIEGVKNLLKKI*
LFGKILWRLAKFIPSVIGHFGGKKKQ
IKWKKIMLRLGKWGIKTGMKHLMNRNTDKKE
NFMSKFKEFIKKTAPKMTKLLQMLTK*

GFGGLLVKMGKVIAPMLISEMPKILNYMKEKM*
LWEKLKSLWEKIKVPIMKIM*
KVNWRKVGKETIKGAAKGAVDGL*
VNWGKWKKIAKEMCPMAKAAMEKACK
KVSFGDMMKKMCKTSPHMKSVCRSIGI
EVKINMDKLKEMMKEICAKIPESIRKKMCPK
KVSFGDMMKKMCKTSPHMKSMCRSIGI
FWGRKRYLKGNRRENRKE
INWKKVVKKMGLCALKAV*
HWVSKLMKVV*
LWPFLISLIPTVIDLARNL*

NWFTSVMKAAGKAAMKAGGKVIAEKAIEATKNKIK*
KVNWRRVGKETIKGAAKGAVDGW*
KVSFGKMVKVMCKTSPHMKSMCRSIGI
INIPWGKIFQWVKKNVM*
GWKTKGAKWIAGKII*
IKWKKIMLRLGKWGMKTGMKHLMNRNTDKKE
IKILDSLRKVVTKLAAKLLPTIKNILK*
GLKEIAGRLLKFVKEKLAGLLK*
IDKKKSRRPWIL
RNVFLPRFMPRI*
EGSLRKITKMFCKSSSHMKSMCQSIGI
RKLVPKI*
AIPPALAEIMKKMTSKLMAQLSRIFGKLKI*
EGSLIYFTKTICKHAAPNMKSICRRIGI
FKLPTWITDLFTKDNMKKMCPMAKAAMEKACK
WMGLAMRLAGGLMSHMMNKKK*
GLRSFWKPFWEKLKPIMIKTL*
NWFTSVMKVVGPTILPLIAEQMPEMVKWIKEKV*
IKFPTWITNVFTKDNMKKMCPMAKAAMEKACK
GWIKWVGKLM*
INWGKLWKVAKIIGKILRSMMARKQKK*
RKVFLPRFISRI*
ALWDTIVSTNRTIHI
WLISLAKYVGPILASYAVDKWPEMKEWATKVAAKI*
KLKGFLKMILKKVISCLLKSV*
FWNRRRGARRSLPIRRATRTDVP
LDPKVIATMQGQNM*
LDAKVIASLQGQGM*
LDPKAIATLQGQSM*
ISPAALASLTGKRNK*
INSVLLASLVGKKTK*
IPPAALASLAGKRK
IPPAALASLAGKRK
CMRSPCPTIGMNR*
KHGQAEKIGIFDQIDKFMDWVMKQTE*
DTPGQAKKIGIFDQIDKGWAWLMKQME*

VEKPGHAKEIGFLERISEMISWLLQKTE*
NIIRVPEFPCPKGQRRDSRGKCRVVMQ
NVIEVPIQCPPGTTYARNHCRDVF

IEGEKGELGPHRLPCPPEYANYCFNGKCVHVVAQDEPGKPCYSCICDKFYIGKRCGTLDLTNPDF
IEGEKGELGPHRLPCPPEYANYCFNGKCVHFVAQDEPGKPCYSCICDKFYIGKRCGTLDLTNPDF
IEGEKGELGPHRLPCPPGYENYCFNGKCVHVVAQDEPGKPCYSCICDEFYIGERCGTLDLTNPGYFLK*
IEGEKRELGPHRLPCPPKLNDENYCFNGKCVHLVAQDEPGKPYYSCICDEFYIGERCGTLDLTNPGYFLKGQS
IEGEKGELRPHRLSCPPKYASYHCFNGKCVHLVAQDEPGKPYYSCICDKFYIGERCDTLDITNPDYFLKGQSST
IRENPYLYIYKTKIKDVVCTNHNNDTFNKVDNVCDDCFNLFRNINLYNDCRQNCFGSEYFPACLEVLLQLDEK

Full prepro

MEIPKFLLIAIIVVGLSGSLTWAHSSAIANPEAIAEAIAEAFANAEAEAEPVAPIVAMAGLGLFAAAVAGLD
MEIPKFLLIAIIVVGLSGSLTWAHSSAIANPEAIAEAIAEAFANAEAEAEPVAPIMAMAGLGLFAAAVAGM
KFLLIAIIVVGLSGSLTWAHSSAIANPEAIAEAIAEAFANAEAEAEPVAPIVAMAGLGLFAVAAAGLDWMS
MEIPKFLLIAIIVVGLSGSLTWAHPLAIADPEAIAEAIAEAFADAEAEAEPIAPIVALLAFSLFSSLPFLHYYVTH
IPKFLLIAIIVVGLSGSLTWAHPLAIADPNAEAVADAEAFADAEAEAEPFLPALPLMLGMMTLPFIHDAATK
TWAHPLAIADPNAEAVADAEAFADAEAEAEPFLPALPLMLGMMTLPFIYDAATKG
MEIPKFLLIAIIVVGLSGSLTWAHPLAIADPNAEAVADAEAFADAEAEAEPILPVLPLMLGMMTLPFIYDAA
KFLLIAIIVVGLSGSLTWAHPLAIADPNAEAVADAEAFADAEAEAEPVLPLLPLMLGMMTLPFIHDAATKG
MEIPKFLLIAIIVVGLSGSLTWAHPLAIADPNAEAVADAEAFADAEAEAEPVLPLLPLMLGMMTLPFIHDA
MEIPKFLLIAIIVVGLSGSLTWAHPLAIADPDAEAIADAEAFADAEAEAFPPLLLLAGLFSLPALQHYIETKWI
MEIPKFLLIAIIVVGFSGSLTWAHPLAIADPDAEAIADAEAFADAEAEAFPPLLLLAGLFSLPALQHYIETNW
EIPKFLLIAIIVVGLSGSLTWAHPLAIADPDAEAIADAEAFADAEAEAEPALPALPLLAFFFTFFPAAQHWLE
MAIADPDAEAIADAEAFADAEAEAEPALPALPLLAFFFYLFPGRTALAREKNNGGLKRRS
MKLSWLSWALAIIFVMAIMDTPIAEAKALASPEAEADADASADAFADAEAKAEAEAALWDTIKKWVAK
MKRIYFLFTIMAIVVLTAQAFSDADAEADAFADPEAMNWGKLKEMAKKMCPMAKAAMEKACK
MKRIYFLFAIIAIVVLTAQAFSEADADAEAEASNMQEIMKKLCTDKKMPAWMCKMLGL
MKLSWLSLALAIIFVMAIMDAPIAEAKAVASPEAKADADASADAFANAEAEAEAEAFWAAVAKWIIPAA
MKRIYFLFAIIAVVVLTTQAFSNADAEAEAWNMEEELKKMIPKEIYEKALNKQNELMACVKNVMKI
MKLSWLSLAFAIIFITVIMDVPKVEAKAVAAPAADADADAFADADAEAKALADAFAFWLKGGRGDGG
MKRIYFLFTIIAIVVLTAQAFSDADAEADAFADPKALNLGKLKEMVKKMCPMAKAAMEKWCK
MKLSWLSWALAITFVMAIMDAPIAEAKALASPEAEADADASADAFANAEAEAEAKAFIGGLIKFIAKFVP
MKLAWLSWALAIIFVMAIMDAPIAEAKAVASPEAEADADASADAFANAEAEAEAEAFWAGFFKTVGT
MKLSWLSWALAIIFVMAIMDAPIAEAKALASPEAEADADASADAFANAEAEAAFKDVMQKIGSGLLKAL
MKRIYFLFAIIAIVVLTAQAFSDADAEADAFADAEAIKWSNLKKWLNKDNMKKMCPVAKAAMEKMCK
IFITVIMDVPKVEAKAVAAPAADADADAFADADAEAKALADAFAFWLKAGRGTGYTG
MKRIYFLFAIIAIVVLTAQAFSDADAEADAFADPEAFNWKKWLNKDNMKKMCPVAKAAMEKMCK
MKLSWLSLALAVIFVMGIMHAPKVEAKAIAAPEADAIGDAWADANADADANADADADADANAAAEAE
MKRIYFLFAIIAIVVLTAQAFSDAEADAFADAEAINWPNWMTKIFTKDNMKKMCPMAKAAMEKACK
MMKLSWLSLAFAIIFITVIMDVPKVEAKAVAAPAADADADAFADADAEAKALADAFAFWLKGGRGDGG
MKLSWLSWALAIIFVMAIMDAPIAEAKALASPEAEADADASADAFADAEADAEAEAEAEAFWGALW
MKLSWLSLALAVIFVMGIMHAPKVKAKAIAAPEADAIADAWADANADADANADADADADANAAAEAE
MKLSWLSLALAIIFVMAIMDAPIAEAKALASPEAEADADASADANADAEAWISKVWKGIKMAGKMLG
MKLSWLSLTLAIIFVMVIMNAPKVEAKAVAASAADADADAFADADAEAEALAVAFALALARNVPRWFT
MKLSWLSWALAIIFVMAIMDAPIAEAKALASPEAEADADASADASADAFADAEAEAEALNWIKMLTGM
MKRIYFLFAIIAIVVLTAQAFSEADADAEAEAFNTEEAKTKIKEMCANNKIPWKRMCEWVNKRA
MKQIYLLFALVVIAVLTAQAHAEATADANSMAVADPLASPEALASAEAEAFAEALADAMADPEARLNLK
MKRIYFLFAIIAIVVLTAQAFSEADAEAEALDVKDLLKKLCAKIPDTIPLKKKLCPK
MKQTYLLFALIAIAVLMVQAHPEAKADPTAVAEAEASAEAEAFADAMAIADAIAMADPEAVDWKGFGS
AEAKALASPEAEADADASADAFADAEAKAEAEAIKISDIIKLAKTVLAALPQNIKDMWKKKGK
MKRIYFLFAIIAIVVLTAQAFSEADADAEAEAFNMEEINAKIKEICARKYSNQKISKWIKIACELVKKYI
MKLSWLSWALAIIFVMTIMDAPIAEAKALASPEAEADADASADAFANAEADAALKDVLKNIGSALLKALS
MKRIYFLFAIIAIVVLTAQAFSEADADAEAELNIDKWKEMMKKMCAKMPEPIKKMCPKL
MKLSWLSWALAIIFVMAIMDAPIAEAKALASPEAEADADASADAFADAEAEAEAIKWGKLLKTAVGLIG
MKLSWLSWALAIIFVMAIMDAPVAEAKALASPEAEADADASADAFADAEAKAEAEAIKISDIIKKAIEGVK
MKLSWLSWALAIIFVMAILDAPIAEAKALASPEAEADADASADAFANAEAEAEAEALFGKILWRLAKFIPS
MKLSWLSWALAIIFVMAIMDAPTAEAKALASPEAKADADASADASADAFAEAEAEAEAIKWKKIMLRLG
MKLSWLSWALAIIFVMAIMDAPIAEAKALAEAEADADASADAFADAEAKAEAEAVANFMSKFKEFIKKT

SWLSWALAIIFVMAIMDAPIAEAKAVASPEAEADADASADADAEAEAKAIAGFGGLLVKMGKVIAPMLI
MKLSWLSWALAIIFVMAIMDAPIAEAKALASPEAEADADASADAFADAEAKAEAEAIALWEKLKSLWEK
MKLSWLSWALAIIFVMAIMDAPIAEAKALASPEAEADADASADASADANADAFADAFADAVAEAEAKV
MKRIYFLFTIIAIVVLTAQAFSDADAEADAFANPEAVNWGKWKKIAKEMCPMAKAAMEKACK
MKRIYFLFAIIAIVVLTAQAFSEADADAEAKVSFGDMMKKMCKTSPHMKSVCRSIGI
MKRIYFLFAIIAIVVLTAQAFSEADADAEVKINMDKLKEMMKEICAKIPESIRKKMCPK
MKQIYFLFAIIAIVVLTAQAFSEADADAEAKVSFGDMMKKMCKTSPHMKSMCRSIGI
MKLSWLSWALAIIFVMAIMDAPIAEAKALASPEAEADADASADASANAFADAEAEAFWGRKRYLKGNR
MSWLSWALAIIFVMAIMDAPIAEAKALASPEAEADADASADASADAFADAEAEAEAINWKKVVKKMGL
LSWALAIIFVMAIMDAPIAEAKALASPEAEADADASADASADAFADAEAEAIAHWVSKLMKVVG
MKLSWLSLALAVIFVMGIMHAPKVEAKAIAAPEADADADAWADANADADANADADADADANADAEA
MKRIYFLFAIIAIVVLTTQTFSEADADADGAFMTAMIKKLCENKRTAADMEPNMCQILNQKRNRRILK
MKLSWLSWALAIIFVMAIMDAPIAEAKALASPEAEADADASADAFADAEAEAIANWFTSVMKAAGKAA
ADASADASADANADAFADAFADAVAEAEAKVNWRRVGKETIKGAAKGAVDGWGKK
MKRIYFLFAIIAIVVLTAQAFSEADADAEAKVSFGKMVKVMCKTSPHMKSMCRSIGI
MKLSWLSWALAIIFVMVIMDAAIAEAKALASPEAEADADANADASADAFADAEAEAEAINIPWGKIFQ
MKLSWLSWALAIIFVMAIMDAPIVEAKALASPEAEADADASADANAGAEAEAEAEAIAGWKTKGAKW
MKLSWLSWALAIIFVMAIMDAPTAEAKALASPEAKADADASADASADAFAEAEAEAEAIKWKKIMLRLG
MMKLSWLSWALAIIFVMAIMDASIAEAKALASPEAEADADASADANADAFADAEAIKILDSLRKVVTKLA
MKLSWLSWALAIIFVMAIMDAPIAEAKALASPEAEADADASADAFANAEAEAGLKEIAGRLLKFVKEKLA
MKQIYFLFAIIAIVVLTAQAFSEADADAEAEAIAIDKKKSRRPWIL
AIIFVMAIMNAPKVEAKAVAAPAADADADAFADADAEAEALAAAFALGLARNVFLPRFMPRIG
MKRIYFLFAIIAIVVLTAQAFSEADADAEAEGSLRKITKMFCKSSSHMKSMCQSIGI
IIFVMAIMNAPKVEAKAVAAPAADADADAFADADAEAEALAAAFALGLARKLVPKIG
MKLSWLSWALAIIFVMAIMDAPIAEAKALASPEAEADADASADAFADANAAAEAAIPPALAEIMKKMTS
MKRIYFLFAIIAIVVLTAQAFSEADADAEAEGSLIYFTKTICKHAAPNMKSICRRIGI
MKRIYFLFAIIAIVVLTAQAFSDADAEADAFADPEAFKLPTWITDLFTKDNMKKMCPMAKAAMEKACK
IFVMGIMHAPKVEAKAIAAPEADADADAIADAWADANADADAEPEAWMGLAMRLAGGLMSHMMN
AIIFVMAIMDAPIAEAKALASPEAEADADANADASADAFADAEAEAEAIAGLRSFWKPFWEKLKPIMIKT
MDAPIAEAKALASPEAEADADASADAFADAEAEAIANWFTSVMKVVGPTILPLIAEQMPEMVKWIKEK
MKRIYFLFAIIAIVVLTAQAFSDAEADAFADAEAIKFPTWITNVFTKDNMKKMCPMAKAAMEKACK
MKLSWLSWALAIIFVMAIMDAPIAEAKALASPEAEADADASADASANAFADAEAGWIKWVGKLMGK
MKLSWLSLALAIIFVMAIMDAPIAEAKALASPEAEADADASADASADASADAFADAEAEAEAINWGKLW
KLSWLSLTLAIIFMTAIMDAPKVETKAVAVSAADADADAFADADAEAEVLADAFALGLARKVFLPRFISRI
MKLSWLSWALAIIFVMAIMDTPIAEAKALASPEAEADADASADAFADAEAKAEAEAALWDTIVSTNRTI
DAPIAEAKALASPEAEADADASADAFADAEAEAIAPWLISLAKYVGPILASYAVDKWPEMKEWATKVAA
VMAIMDAPIAEAKALASPEAEADADASADASADAFANAEAKAKLKGFLKMILKKVISCLLKSVG
IMDAPIAEAKALASPEAEADADASADASANAFADAEAEAFWNRRRGARRSLPIRRATRTDVP
MKTIMLTISIAMLMMTMASLVLARPDALDPKVIATMQGQNMGK
MKTMMLTISIAMLMMTMASLVLARPDALDAKVIASLQGQGMGK
MKTMMLTISIAMLMMTMASLVLTRPDALDPKAIATLQGQSMGK
MKTLKLIISIAIIMAAMAVLTSAKPDAISPAALASLTGKRNKGK
MKTLKLIISIAIIMAAMAVLTSAKPDAINSVLLASLVGKKTKGK
MKTLIISIAIIMAAMAVLTSAKPDAIPPAALASLAGKRK
MKTLIISIAIIMAAMAVLTSAKPDAIPPAALASLAGKRK
MKTFLLLLGVIVMLTITMLPIANAKAEPCMRSPCPTIGMNRG
MKVTYAILLVAVITVVMVPNIMADAEAEAKHGQAEKIGIFDQIDKFMDWVMKQTEG
MKVTYAILLVAVITVVMVPNIMADAEAEADTPGQAKKIGIFDQIDKGWAWLMKQMEG

MKTTYAILLVAVIAVVMVSNIMADAEAVEKPGHAKEIGFLERISEMISWLLQKTEG
MKNNISIFAVYLIITFLLISTFMTMVTEANIIRVPEFPCPKGQRRDSRGKCRVVMQ
MRDSHISMFTVYLMATFLLISTFMAMVISESNVIEVPIQCPPGTTYARNHCRDVF
MRPNVLISILFVLGVILSVWRPGDAAMALPHSRTKRTIYIITHKPKKSKHPIIITGKPAKITDIYLRR
MKDSYISIVIAYLMVTFILVSSMPIEGEKGELGPHRLPCPPEYANYCFNGKCVHVVAQDEPGKPCYSCICDK
MKDSYISIVIAYLMVTFILVSSMPIEGEKGELGPHRLPCPPEYANYCFNGKCVHFVAQDEPGKPCYSCICDK
MKDSYISIVIAYLMVTFILVSSMPIEGEKGELGPHRLPCPPGYENYCFNGKCVHVVAQDEPGKPCYSCICDE
MKDSYISIVIAYLMVTFILVSSMPIEGEKRELGPHRLPCPPKLNDENYCFNGKCVHLVAQDEPGKPYYSCICD
MKDSYISIVIAYLMVTFILVSSMPIEGEKGELRPHRLSCPPKYASYHCFNGKCVHLVAQDEPGKPYYSCICDK
MLFRLILLFIFVYYILLTSTIRENPYLYIYKTKIKDVVCTNHNNDTFNKVDNVCDDCFNLFRNINLYNDCRQN

DWLSKKVG
DWLAKKVG
SKHVG
HTTEKP
G

TKG

AKG
NG
ING
KKIMAG

VLPLVKNALKGK

LSYAPDVIEWMKNKLG

KILPFFQKKN
AMLPYMIDKMPEMAKWMQEKIKQIG
SFIGKRRK

EAFLPFLIPVIASLVSSLKG

VRLAKIIPSAIGGLFGGKKKQ
AILPALIPVFASLLPWAIKKFG

PRLG
IGKWVAKKAIGHFMKKKNE

GIGWKSRGPSFRAGTDPSFGG

KIKRFFKKHKSTIAKTVLKIVTTVLPLVLGK

FFGK

NLLKKIGKK
VIGHFGGKKKQ
KWGIKTGMKHLMNRNTDKKE
APKMTKLLQMLTKGK

SEMPKILNYMKEKMG
KVPIMKIMG
NWRKVGKETIKGAAKGAVDGLGKK

RENRKE
CALKAVG

EALWPFLISLIPTVIDLARNLG

MKAGGKVIAEKAIEATKNKIKG

VVKKNVMG
AGKIIG
KWGMKTGMKHLMNRNTDKKE
AKLLPTIKNILKGK
GLLKGK

KLMAQLSRIFGKLKIGK

KKKG
LG
VG

VKVAKIIGKILRSMMARKQKKG
G
HI
KIG

FYIGKRCGTLDLTNPDF
FYIGKRCGTLDLTNPDF
FYIGERCGTLDLTNPGYFLKG
EFYIGERCGTLDLTNPGYFLKGQSSTQSSI
FYIGERCDTLDITNPDYFLKGQSSTQSSI
CFGSEYFPACLEVLLQLDEKQKHLEWVTQLHNGSTTNITWS
