# Supplementary material for: Intra-colony venom diversity contributes to maintaining eusociality in a cooperatively breeding ant
Source: BMC Biol. 2023 Jan 8;21:5. doi: 10.1186/s12915-022-01507-9 (PMC9827630; doi:10.1186/s12915-022-01507-9)
Supplement: Supplementary file 4 — Additional file 4: Figure S1. Activity of Rm1a and Rm4a. Figure S2. Cytotoxicity of R. metallica aculeatoxin peptides. Figure S3. Gene structures of some ant aculeatoxins. Figure S4. Presence of aculeatoxin alleles among workers from the same R. metallica colony. Figure S5. Phylogeny of R. metallica aculeatoxins estimated from full-length or only signal- and propeptide domains. Table S4. Recombination analysis of R. metallica clades 1–3.Table S5. Primers used to amplify aculeatoxins from R. metallica genomic DNA. [file 12915_2022_1507_MOESM4_ESM.pdf]

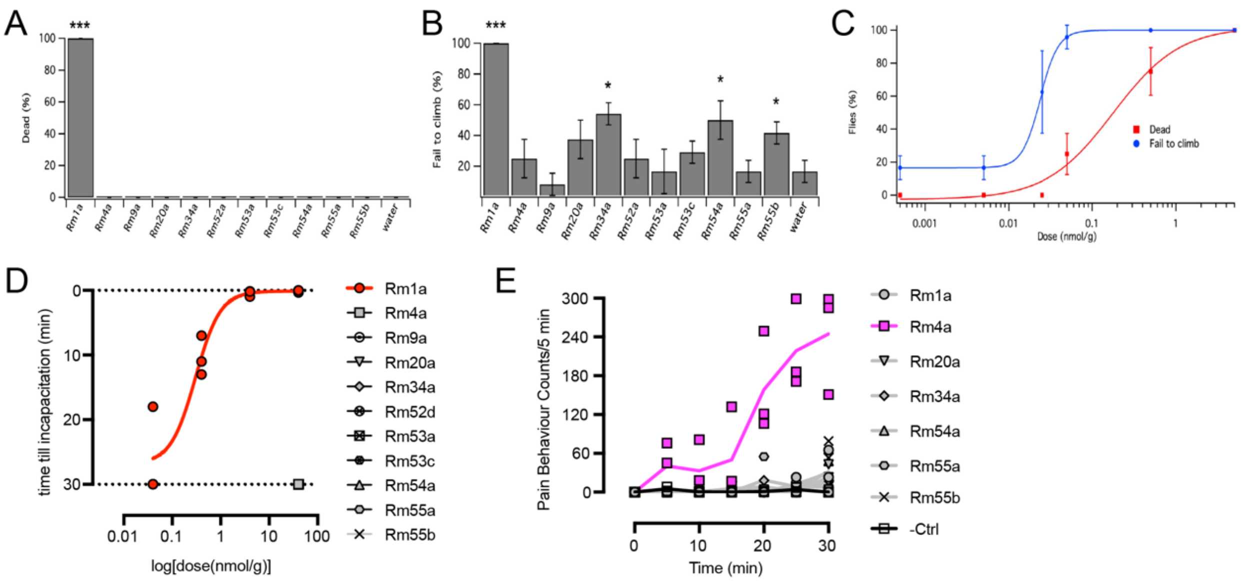

Figure S1. Activity of Rm1a and Rm4a. (A) Injection of 5 nmol/g Rm1a, but no other peptides tested, caused instant death in 100 ± 0% of *D. melanogaster*. (B) Locomotion-impairing effect of 5 nmol/g of each peptide as measured using a *D. melanogaster* climbing assay 5 min after injection. (C) Dose-response curve for paralytic and lethal effects of Rm1a measured at 5 min after injection in *D. melanogaster*. Data points and error bars represent mean ± SD respectively. Asterisks indicate a significant difference compared to negative control using a one-tailed unpaired Student's t-test; *, $P < 0.05$, *** $P < 0.001$. (D) Insecticidal effect against crickets ($n = 3$; see Figure 4b). (E) Spontaneous pain behaviours in mice ($n = 3$; see Figure 4c).

A

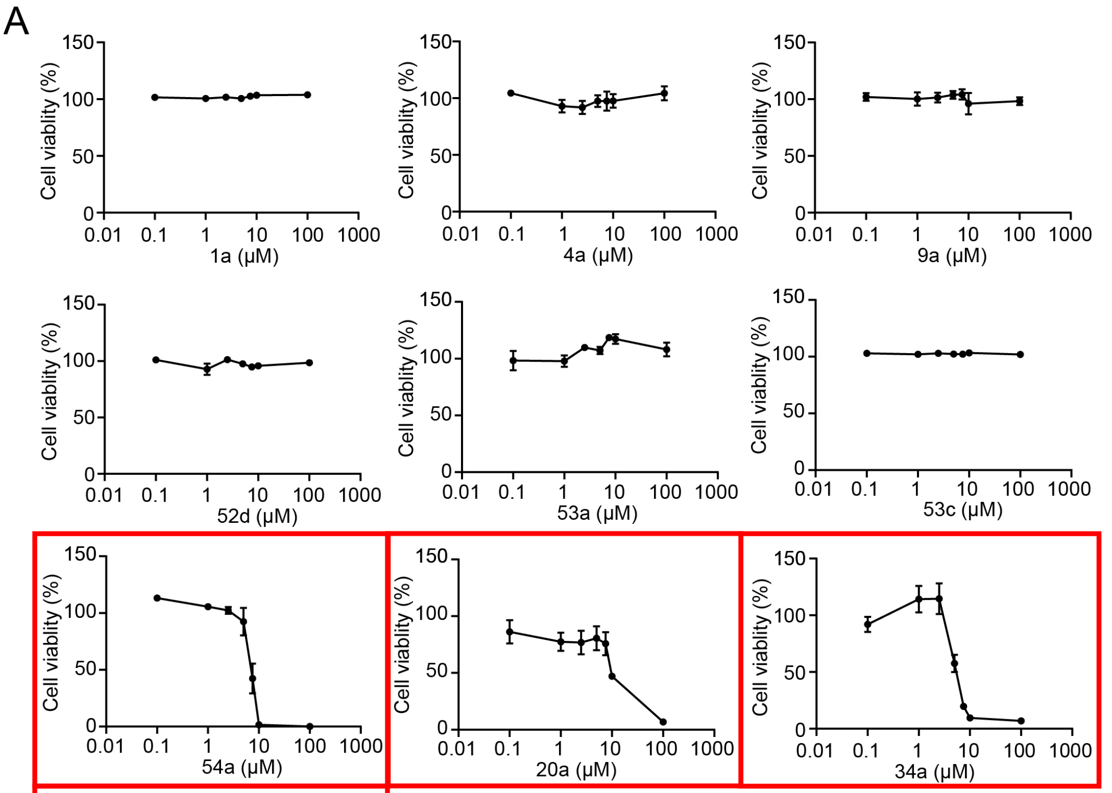

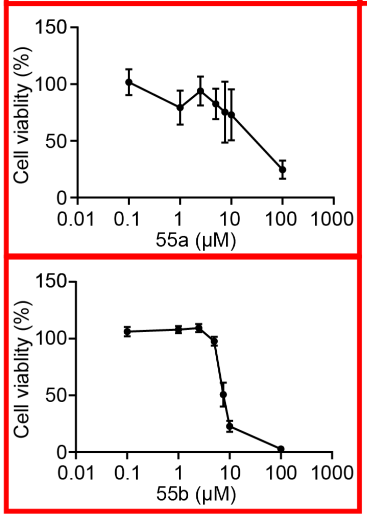

B

Peptide	HAP1	MM96L	HEK293T
55b	✓✓✓	✓✓✓	✓✓✓
55a	✓✓✓	✓✓✓	✓✓✓
52d	✗	✓(2%)	✗
4a	✗	✓(1%)	✓(12%)
53c	✗	✓(1%)	✗
20a	✓✓✓	✓✓✓	✓✓✓
34a	✓✓✓	✓✓✓	✓✓✓
53d	✗	✗	✓ (16%)
54a	✓✓✓	✗	✓✓✓
9a	✗	✓✓✓	✓✓ (70%)
1a	✗	?	?

Figure S2. Cytotoxicity of *R. metallica* aculeatoxin peptides. (a) Cytotoxicity of peptides at various concentrations in HAP1 cells. (b) Summary of peptide cytotoxicity in the three cell lines used. Colouration of boxes indicate cytotoxicity (dark orange = very toxic, light orange = mildly toxic, grey = no toxicity observed).

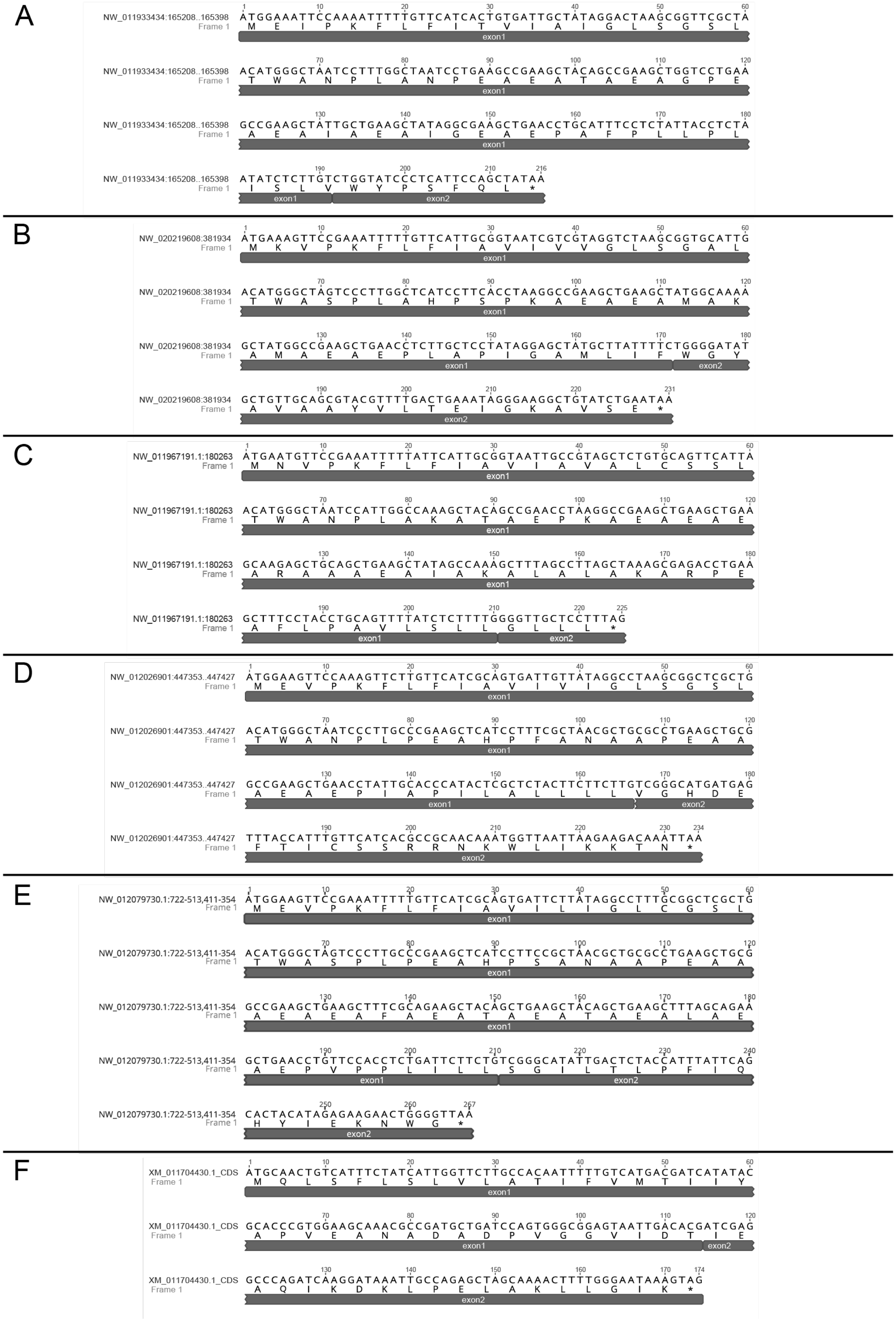
A
NW_011933434:165208..165398
Frame 1
ATGGAAATTCCAAAATTTTTGTTCATCACTGTGATTGCTATAGGACTAAGCGGTTCGCTA
M E I P K F L F I T V I A I G L S G S L
ACATGGGCTAATCCTTTGGCTAATCCTGAAGCCGAAGCTACAGCCGAAGCTGGTCCTGAA
T W A N P L A N P E A E A T A E A G P E
GCCGAAGCTATTGCTGAAGCTATAGGCGAAGCTGAACCTGCATTTCCTCTATTACCTCTA
A E A I A E A I G E A E P A F P L L P L
ATATCTCTTGTCTGGTATCCCTCATTCCAGCTATAA
I S L V W Y P S F Q L *
exon1
exon2
B
NW_020219608:381934
Frame 1
ATGAAAGTTCCGAAATTTTTGTTCATTGCGGTAATCGTCGTAGGTCTAAGCGGTGCATTG
M K V P K F L F I A V I V V G L S G A L
ACATGGGCTAGTCCCTTGGCTCATCCTTCACCTAAGGCCGAAGCTGAAGCTATGGCAAAA
T W A S P L A H P S P K A E A E A M A K
GCTATGGCCGAAGCTGAACCTCTTGCTCCTATAGGAGCTATGCTTATTTTCTGGGGATAT
A M A E A E P L A P I G A M L I F W G Y
GCTGTTGCAGCGTACGTTTTGACTGAAATAGGGAAGGCTGTATCTGAATAA
A V A A Y V L T E I G K A V S E *
exon1
exon2
C
NW_011967191.1:180263
Frame 1
ATGAATGTTCCGAAATTTTTATTCATTGCGGTAATTGCCGTAGCTCTGTGCAGTTCATTA
M N V P K F L F I A V I A V A L C S S L
ACATGGGCTAATCCATTGGCCAAAGCTACAGCCGAACCTAAGGCCGAAGCTGAAGCTGAA
T W A N P L A K A T A E P K A E A E A E
GCAAGAGCTGCAGCTGAAGCTATAGCCAAAGCTTTAGCCTTAGCTAAAGCGAGACCTGAA
A R A A A E A I A K A L A L A K A R P E
GCTTTCCTACCTGCAGTTTTATCTCTTTTGGGGTTGCTCCTTTAG
A F L P A V L S L L G L L L *
exon1
exon2
D
NW_012026901:447353..447427
Frame 1
ATGGAAGTTCCAAAGTTCTTGTTCATCGCAGTGATTGTTATAGGCCTAAGCGGCTCGCTG
M E V P K F L F I A V I V I G L S G S L
ACATGGGCTAATCCCTTGCCCGAAGCTCATCCTTTCGCTAACGCTGCGCCTGAAGCTGCG
T W A N P L P E A H P F A N A A P E A A
GCCGAAGCTGAACCTATTGCACCCATACTCGCTCTACTTCTTCTTGTCGGGCATGATGAG
A E A E P I A P I L A L L L L V G H D E
TTTACCATTTGTTCATCACGCCGCAACAAATGGTTAATTAAGAAGACAAATTAA
F T I C S S R R N K W L I K K T N *
exon1
exon2
E
NW_012079730.1:722-513,411-354
Frame 1
ATGGAAGTTCCGAAATTTTTGTTCATCGCAGTGATTCTTATAGGCCTTTGCGGCTCGCTG
M E V P K F L F I A V I L I G L C G S L
ACATGGGCTAGTCCCTTGCCCGAAGCTCATCCTTCCGCTAACGCTGCGCCTGAAGCTGCG
T W A S P L P E A H P S A N A A P E A A
GCCGAAGCTGAAGCTTTCGCAGAAGCTACAGCTGAAGCTACAGCTGAAGCTTTAGCAGAA
A E A E A F A E A T A E A T A E A L A E
GCTGAACCTGTTCCACCTCTGATTCTTCTGTCGGGCATATTGACTCTACCATTTATTCAG
A E P V P P L I L L S G I L T L P F I Q
CACTACATAGAGAAGAACTGGGGTTAA
H Y I E K N W G *
exon1
exon2
F
XM_011704430.1_CDS
Frame 1
ATGCAACTGTCATTTCTATCATTGGTTCTTGCCACAATTTTTGTCATGACGATCATATAC
M Q L S F L S L V L A T I F V M T I I Y
GCACCCGTGGAAGCAAACGCCGATGCTGATCCAGTGGGCGGAGTAATTGACACGATCGAG
A P V E A N A D A D P V G G V I D T I E
GCCCAGATCAAGGATAAATTGCCAGAGCTAGCAAAACTTTTGGGAATAAAGTAG
A Q I K D K L P E L A K L L G I K *
exon1
exon2

23

Figure S3. Gene structures of some ant aculeatoxins. The prepropeptide of Rm4a was searched against the ant (Formicidae) part of the NCBI RefSeq Genome Database with tblast using default parameters. Hits that contained full open reading frames (ORFs) included (A) NW_011933434 from *Pogonomyrmex barbatus,* (B) NW_020219608 from *Themnothorax curvispinosus*, (C) NW_011967191 from *Vollenhovia emeryi*, and (D) NW_012026901, (E) NW_012079730.1, and (F) XM_011704430 from *Wasmannia auropunctata.* Only the transcript ORFs are shown for each gene, with the translated sequence and corresponding exons displayed below.

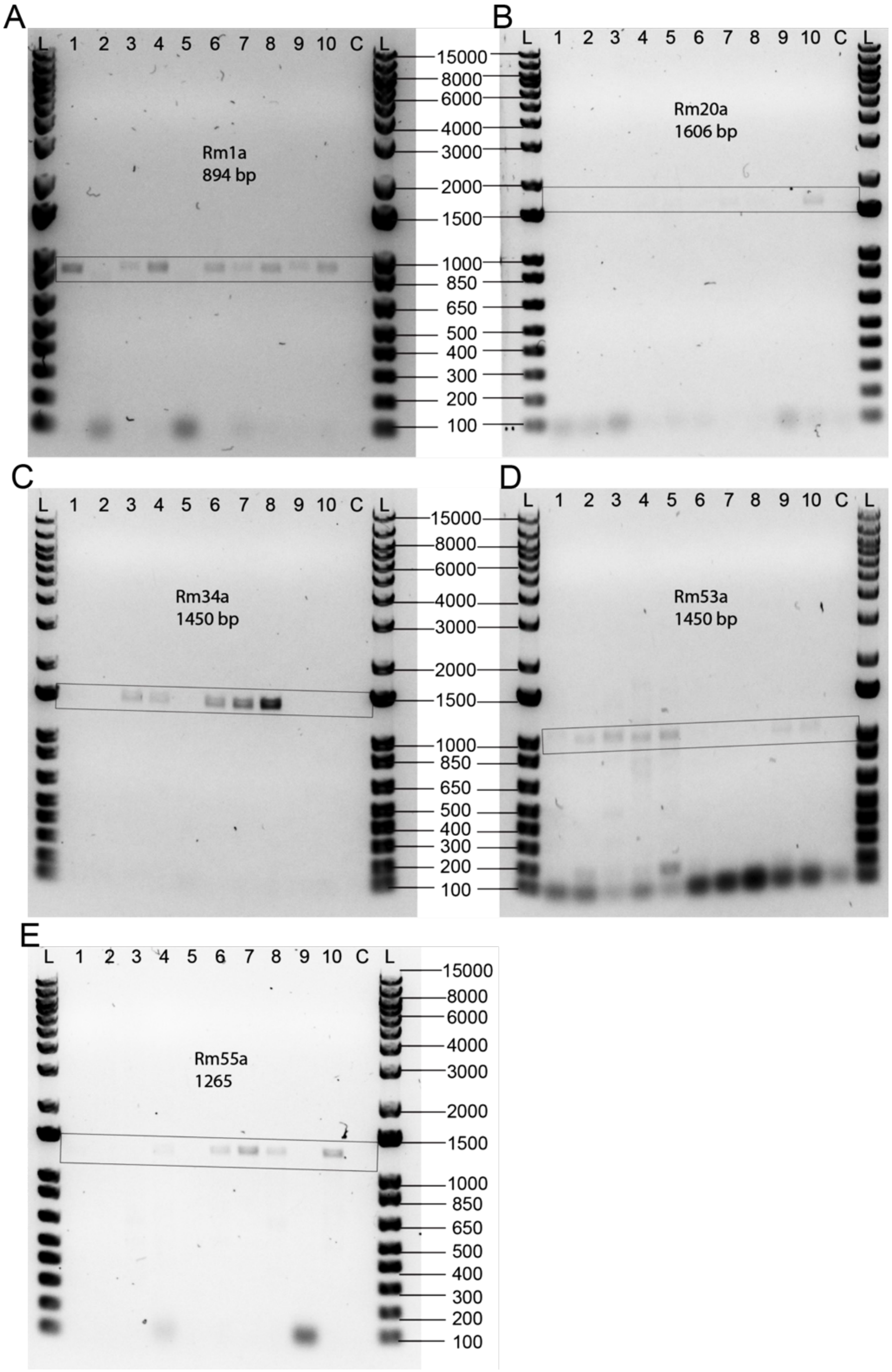

Figure S4. Presence of aculeatoxin alleles among workers from the same *R. metallica* colony. Full gel images of those shown in Figure 6D of the main manuscript. PCR products were separated on 1% agarose gels with SYBR safe DNA stain ((Thermo Fisher, Waltham, MA, USA)) and imaged on a Gel Doc imager (Bio-Rad, USA). Brightness and contrast were optimized to maximize visualization of bands. DNA standards (1Kb Plus DNA Ladder (Thermo Fisher, Waltham, MA, USA)) and their corresponding length (bp) are shown in the left- and right-most lanes (L), individual ants are labelled 1–10, while C represents negative control.

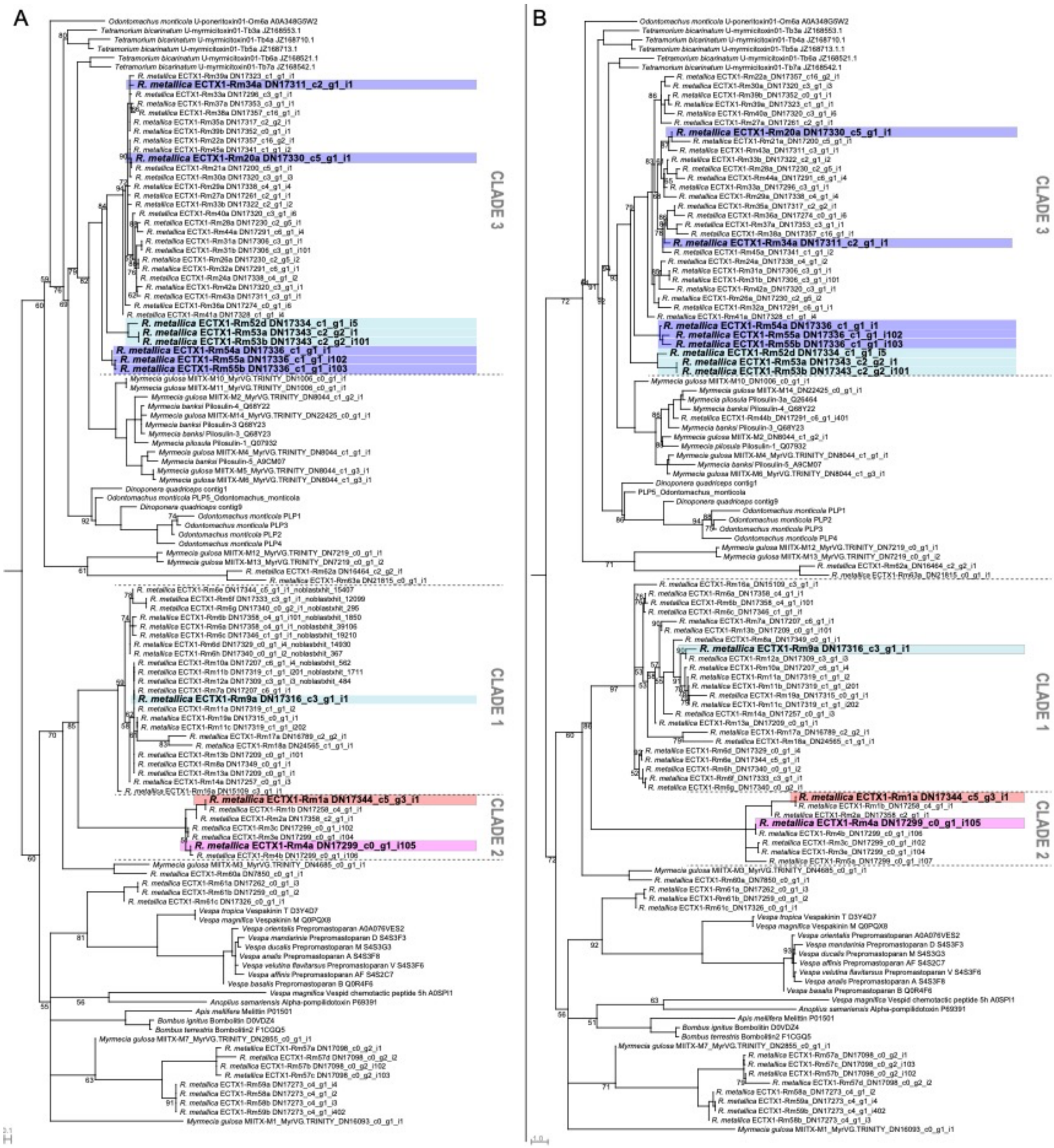

Figure S5. Phylogeny of *R. metallica* aculeatoxins estimated from full-length or only signal- and propeptide domains. Because our Genetic Algorithm for Recombination Detection analysis revealed evidence of breakpoints between the pro- and mature peptide domains in all clades, we performed phylogenetic analyses based on alignments of either the full coding regions or just the signal- and propeptide domains. These analyses returned near-identical topologies, suggesting the overall topology of the phylogeny shown in Figure 3 is robust. Sequence alignments can be found in SI Data.

Table S4. Recombination analysis of *R. metallica* clades 1–3*.* The Genetic Algorithm for Recombination Detection (GARD) revealed evidence of breakpoints between the pro- and mature peptide domains in all clades. However, for Clade 1, comparing the AIC_c score of the best fitting GARD model, that allows for different topologies between segments, to that of the model that assumes the same tree for all the partitions inferred by GARD, but allows different branch lengths between partitions, suggests that because the multiple tree model could not be preferred over the single tree model by an evidence ratio of 100 or greater, some or all of the breakpoints may reflect rate variation instead of topological incongruence. For clades 2 and 3, the multiple tree model could be preferred over the single tree model by an evidence ratio of 100 or greater, meaning at least of one of the breakpoints reflects a true topological incongruence.

**Clade**	**BPs**	**AIC_c**	**Δ AIC_c**	**Segments**
1	0	3910.94		1 - 261
	1	3772.47	138.47	1 - 8384 - 261
2	0	2341.34		1 - 258
	1	2120.31	221.03	1 - 171172 - 258
	2	2117.29	3.02	1 - 153154 - 202203 - 258
3	0	12561.48		1 - 345
	1	11224.96	1336.53	1 - 195196 - 345

Table S5. Primers used to amplify aculeatoxins from *R. metallica* genomic DNA.

**Peptide**	**Forward primer**	**Reverse primer**
Rm1a	GTAGCGCCTATCGTGGCAAT	TTCGACAGCCAGTCCAAAC
Rm20a	AAAGGCAGAGGCTGAGGC	CACTAGTGGTAATACTTTTGCTACC
Rm34a	GAGGCTGAGGCCAAAGCTTTTAT	GCAAAATCTTCGGCACGAACT
Rm53a	CTTTGCCGATGCAGATGCCG	CTCCTCTCCCTCCTTTCAACCA
Rm55a	ATGCAAATGCCGCTGCAGAA	CACTAAGGATGCTATCACCGGA
DPP-4	GGGACGACAACGTGCATTTC	TGTGTGATAGAGGTGCGGAG
